# Stabilizing Effect of a 4c/6e Hypervalent Bond in Dinitrodiphenyl Disulfides and Their Thermochemical Properties: Experimental and Computational Approach

**DOI:** 10.1021/acs.jpca.3c01624

**Published:** 2023-06-27

**Authors:** Henoc Flores, Fernando Ramos, Julio M. Hernández-Pérez, Juan M. Solano-Altamirano, E. Adriana Camarillo, Jacinto Sandoval-Lira

**Affiliations:** † Facultad de Ciencias Químicas, Benemérita Universidad Autónoma de Puebla, 14 sur y Av. San Claudio, C.P. 72570 Puebla Pue., Mexico; ‡ Departamento de Ingeniería Ambiental, Instituto Tecnológico Superior de San Martín Texmelucan, C.P. 74120 San Martín Texmelucan, Pue., Mexico

## Abstract

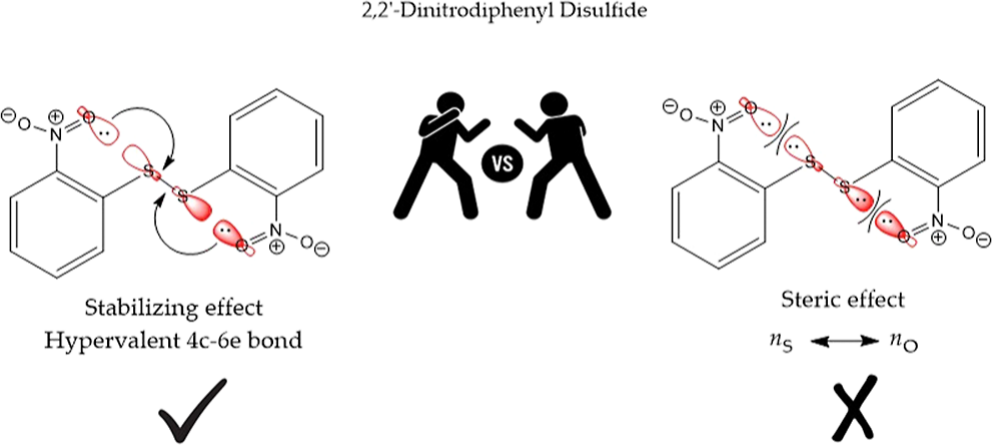

Thermochemical properties and intramolecular interactions of 2,2′-dinitrodiphenyl disulfide (2DNDPDS) and 4,4′-dinitrodiphenyl disulfide (4DNDPDS) were determined and analyzed. Their standard molar formation enthalpies in the gas phase (Δ_f_*H*_m_^°^(g)’s) were experimentally determined; theoretically, they were computed using the G4 composite method and atomization reactions. Specifically, Δ_f_*H*_m_^°^(g)’s were obtained by combining formation enthalpies in the condensed phase and enthalpies of phase change. Formation enthalpies in the condensed phase were determined experimentally through combustion energies, which in turn were found by means of a rotatory bomb combustion calorimeter. Sublimation enthalpies were derived from thermogravimetric experiments, measuring the rate of mass loss and using Langmuir and Clausius–Clapeyron equations. Fusion enthalpies and heat capacities of the solid and liquid phases were measured as functions of temperature by differential scanning calorimetry, and the heat capacities of the gas phase were calculated via molecular orbital calculations. Theoretical and experimental Δ_f_*H*_m_^°^(g)’s differed by <5.5kJ·mol^–1^, and isomerization enthalpies are discussed. In addition, using theoretical tools [natural bond orbitals (NBO) and quantum theory of atoms in molecules (QTAIM)], intramolecular interactions were analyzed. An uncommon hypervalent four-center six-electron interaction of type O···S–S···O was found in 2DNDPDS. This hypervalent interaction, in addition to the extent of conjugation between the aryl and NO_2_ moieties and the formation of intramolecular C–H···S hydrogen bonds, counteracts the repulsion caused by steric repulsions. Hydrogen bonding was confirmed through geometric parameters as well as QTAIM.

## 1 Introduction

Organic disulfides of the R–S–S–R′ form, where R and R′ are aryl or alkyl groups, play a significant role in biochemistry, since their presence or absence determines the functionality of some proteins.^1^

These types of disulfides play essential roles as auxiliaries in synthetic sequences and are widely used as intermediates in the synthesis of pharmaceutical products,^2^ acylation reactions of anhydrides,^3^ and in the synthesis of phenanthrene derivatives^4^ and high angular tension molecules such as sulfur tetrahedranes^5^ and thiocarbamates.^6^ An important use of these compounds is found as well in the synthesis of sulfenylindoles, which are structures of biological and pharmaceutical importance for treating heart diseases, allergies, cancer, HIV, and obesity.^7^ Recently, it has been discovered that some aryl disulfides have activity against leishmaniasis.^8^

Despite the use of disulfides in organic synthesis and their applications in biological and medical sciences, very few reports about their thermochemical properties exist. Certainly, Mackle and Mayrick^9^ reported several thermochemical properties of alkyl and aryl sulfides and diphenyl disulfide; however, the data did not include information on nitro derivatives. Mackle and McClean^10^ reported the enthalpy of formation in the gas phase of 2,2,5,5-tetramethyl-3,4-dithiahexane. Hubbard et al.^11^ experimentally determined the enthalpy of combustion and enthalpy of vaporization of 2,3-dithiabutane and 3,4-dithiahexane. Recently, we have reported several thermochemical properties of diphenyl disulfide and 2,2′- and 4,4′-dinitrodiphenyl disulfides,^12^ including enthalpies of formation in the solid and liquid phases, fusion temperatures, and phase-change enthalpies. Paulechka and Kazakov^13^ used the LCCSD(T) (local coupled cluster with single, double, and perturbative triple excitations) protocol to theoretically estimate the enthalpy of formation of some disulfides: 2,3-dithiabutane, 3,4-dithiahexane, 2,2,5,5-tetramethyl-3,4-dithiahexane, diphenyl disulfide, and 2,2′- and 4,4′-diaminodiphenyl disulfides.

**Figure 1:**
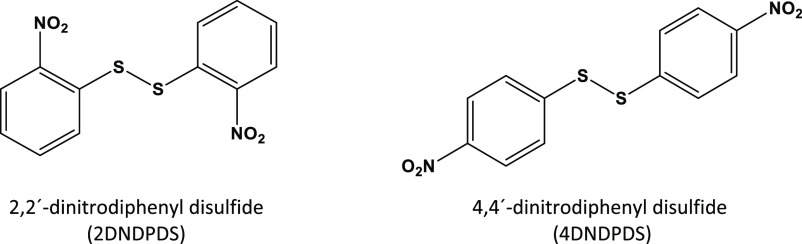
Molecular structures of diphenyl disulfides studied in the present work.

In order to continue studying the energetics of substituted diphenyl disulfides, in the present work, we study 2,2′-dinitrodiphenyl disulfide (2DNDPDS) and 4,4′-dinitrodiphenyl disulfide (4DNDPDS). Figure 1 shows the molecular structures of these compounds.

We used differential scanning calorimetry analysis to determine properties such as purities, fusion temperatures, enthalpies of fusion, and heat capacities of the solid and liquid phases. Specific combustion energies were obtained through a rotatory bomb combustion calorimeter. Mass loss rates were measured as a function of temperature using thermogravimetry; sublimation enthalpies were obtained from this relationship and by applying the Clausius–Clapeyron and Langmuir equations. In addition, we used the G4 composite method and atomization reactions to compute enthalpies of formation in the gas phase. Natural bond orbitals (NBO) and quantum theory of atoms in molecules (QTAIM) were used to theoretically analyze weak intramolecular interactions (see Section 3 for further details).

Finally, the so-obtained experimental and theoretical thermochemical data were used to discuss the relative stability between diphenyl disulfides.

## 2 Experimental Section

### 2.1 Materials and Purity Control

The disulfides studied were obtained from Sigma-Aldrich Co. The provider reports a molar fraction of 0.99 for 2DNDPDS [CAS 1155-00-6]; however, no molar fraction is reported for 4NDFDS [CAS 100-32-3]. Purities were tested through differential scanning calorimetry. The disulfides were subsequently purified by successive recrystallizations using, as the solvent, mixtures of ethanol-ethyl acetate for 2DNDPDS and acetone-ethyl acetate for 4DNDPDS. To eliminate solvent traces and to avoid potential water absorption, the crystals obtained from the recrystallization process were crushed and stored at 353.15 K for 24 h. Subsequently, the samples were stored under a nitrogen atmosphere. This method yielded final molar fractions > 0.999. The initial and final purities of the samples for each compound are presented in Table 1.

**Table 1:** Chemical Data, Sources, and Purities of 2DNDPDS and 4DNDPDS, Indium, and Benzoic Acid

compound	CAS number	source	initial mole fraction purity	purification method	final mole fraction purity^a^	analysis method
2DNDPDS(s)	1155-00-6	Aldrich	0.99	recrystallization	0.9996 ± 0.0002	DSC
4DNDPDS(s)	100-32-3	Aldrich	no reported	recrystallization	0.9990 ± 0.0002	DSC
indium(s)	7440-74-6	PerkinElmer	0.9999929	none	0.9999 ± 0.0001	DSC
benzoic acid(s)	65-85-0	NIST	0.999996	none		

a Numbers that follow the “±” symbol are expanded uncertainties calculated from standard uncertainties of Type A. They include contributions from calibration and have a confidence level of 0.95 and coverage factor *k* = 3.18 (assuming a *t*-student distribution).

Molar fractions and melting temperatures were determined via differential scanning calorimetry (DSC). To this end, the van’t Hoff equation and the fractional fusion method were applied to each melting thermogram. Thereafter, the area under the melting curve was computed to derive the respective enthalpy of fusion.^14,15^ For these experiments, DSC Q2000 equipment from TA Instruments was used, which was calibrated for temperature and heat flow using high-purity indium.^16^ Heating rates of 1 K·min^–1^ and nitrogen flow of 50 cm^3^·min^–1^ were used. The uncertainty associated with each property obtained through DSC corresponds to the expanded uncertainty with a coverage factor *k* = 3.18 and a confidence level of 0.95. Contributions stemming from the equipment’s calibration were included in this uncertainty.

The densities of diphenyl disulfides were taken from the literature, and they correspond to 1.545 g·cm^–3^^17^ and 1.556 g·cm^–3^^18^ for 2DNDPDS and 4DNDPDS, respectively. Atomic weights of elements follow the 2016 IUPAC recommendation.^19^

### 2.2 Heat Capacity

Solid- and liquid-phase heat capacities were determined using a DSC 8000 PerkinElmer differential scanning calorimeter and applying the two-step method. High-purity sapphire was used as reference material.^16^ The heating ramps were 10 K·min^–1^, and a 20 cm^3^·min^–1^ nitrogen flow was applied. Samples of approximately 10 mg were used. A detailed description of the method for obtaining these values has been described previously.^20^

The solid-phase heat capacity of 2DNDPDS was determined to be from 283.15 to 453.15 K, and that of 4DNDPDS was determined to be from 288.15 to 423.15 K, respectively. The liquid-phase heat capacities were determined over the intervals (485.15–506.15) K and (473.15–506.15) K for 2DNDPDS and 4DNDPDS, respectively.

The sample masses used for fusion and heat capacity experiments were weighed on airtight aluminum cells using a Mettler Toledo UMX2 balance, which has a 0.1 μg sensitivity.

### 2.3 Combustion Calorimetry

Combustion experiments were carried out using a rotatory bomb combustion calorimeter, which has a Parr 1004C combustion bomb; this bomb is internally coated with platinum and has an internal volume of 0.348 dm^3^. The calorimeter was calibrated by burning benzoic acid (NIST Standard Reference Material 39j), which is known to have a specific combustion energy of −26414 J·g^–1^. From the calibration experiments, the energy equivalent of the calorimeter was determined to be ε(calor) = 14362.7 ± 2.2 J·K^–1^;^21^ the uncertainty in ε is expressed as the standard uncertainty.

A few combustion experiments showed incomplete combustion, which is not very surprising, as it is known that some aryl disulfides (such as those studied in this work) may act as flame retardants.^22^ Hence, in order to evade this problem, benzoic acid (NIST Standard Reference Material 39j) was used as auxiliary material. The diphenyl disulfide samples were surrounded with the auxiliary material and subsequently shaped as tablets of approximately 1 g. Each pellet was burned in the presence of 10 cm^3^ of deionized water and of oxygen at a pressure of 3.04 MPa. In order to promote the complete conversion of sulfur to sulfuric acid, atmospheric air was not removed. Wicks were made from threads of cotton, whose combustion energy is (−16954.1 ± 3.1) J·g^–1^, where the associated uncertainty corresponds to the standard uncertainty. The solution obtained after each combustion experiment was titrated with sodium hydroxide. Further details of this experimental technique have been described in a previous work.^12^

In order to calculate the specific combustion energy of each disulfide at the standard state, Washburn corrections were applied, following the procedure described by Hubbard et al. for sulfurous compounds.^23^ The pressure coefficient of massic energy, (∂*u*/∂*p*)_*T*_, at 298.15 K was considered to be −0.2 J·g^–1^·MPa^–1^, which is a typical value of organic compounds.^24^ The above procedure has been successfully applied in previous combustion calorimetry measurements involving sulfurous compounds.^12,21,25^ Combustion energies were calculated using the NIST-recommended web tool.^26^ The uncertainties are given as expanded uncertainties at a coverage factor *k* = 1.96 and a confidence level of 0.95, and they include the uncertainty stemming from the calibration experiments, as well as from uncertainties in the combustion energies of auxiliary materials and the diphenyl disulfides. Enthalpies of formation in the crystalline phase were calculated using the formation enthalpies of CO_2_ (g), H_2_O (l), and H_2_SO_4_·115H_2_O (aq) as (−393.51 ± 0.13) kJ·mol^–1^, (−285.830 ± 0.042) kJ·mol^–1^ and (−887.811 ± 0.044) kJ·mol^–1^, respectively.^27,28^

### 2.4 Thermogravimetric Analysis

Equation 1, below, was used to determine the vaporization and sublimation enthalpies through thermogravimetry, and it is obtained by combining the Langmuir and Clausius–Clapeyron equations.
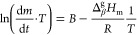
1In eq 1, d*m*/d*t* is the rate of mass loss at temperature *T*, Δ_β_^g^*H*_m_ is the enthalpy of vaporization (β = l) or of sublimation (β = s), *R* is the ideal gas constant, and *B* is a constant that includes the area (*A*) exposed to the phase change, the molar mass *M*, the evaporation coefficient α, and the factor 2π. Here, it is important to remark that eq 1 includes the diffusional effect (*D*) of the gaseous phase, as suggested by Pieterse and Focke,^29^ although it is hidden in the constant *B*. Furthermore, the use of eq 1 has been tested in our laboratory over a series of standard reference compounds and several organic compounds.^12,20,25,30,31^

A TA Instruments Q500 device was used to perform the thermogravimetric experiments. This device has a balance with a maximum load capacity of 1 g and a sensitivity of ±0.1 μg, and it was calibrated with certified NIST masses of 100 and 1000 mg. The device’s thermocouple (which has a sensitivity of ±0.1 K) was calibrated using the Curie temperatures of alumel and nickel as references. The equipment has a vertical furnace and a horizontal purge gas system. On the furnace’s interior is a platinum cell, where samples are deposited. Masses of compounds between 10 and 20 mg were used, and heating ramps of 10 K·min^–1^ were applied from room temperature to 500 and 550 K, for 2DNDPDS and 4DNDPDS, respectively. A nitrogen atmosphere of 100 cm^3^·min^–1^ was used.

Throughout the experiments, the vapor of each sample was condensed out from the heating furnace of the equipment, and it was subsequently analyzed by DSC; no thermal effect, in addition to the melting, was observed. The previous procedure showed that the diphenyl disulfides did not suffer any decomposition, not even at the highest temperatures used in TGA experiments. The uncertainty in the enthalpy of vaporization of each experiment was calculated as a combined uncertainty *u*_comb_, which takes into account the uncertainties in the slope, temperature, and d*m*/d*t*.

Mean vaporization enthalpies were computed as weighted averages through the rule . Here, μ represents the weighted average vaporization enthalpy, *x*_*i*_ stands for an individual enthalpy of vaporization (obtained from the *i*-th experimental run), and *u*_comb,*i*_ is its associated uncertainty. The uncertainty of the weighted average was computed as  with *N* = 4.^32,33^

**Figure 2:**
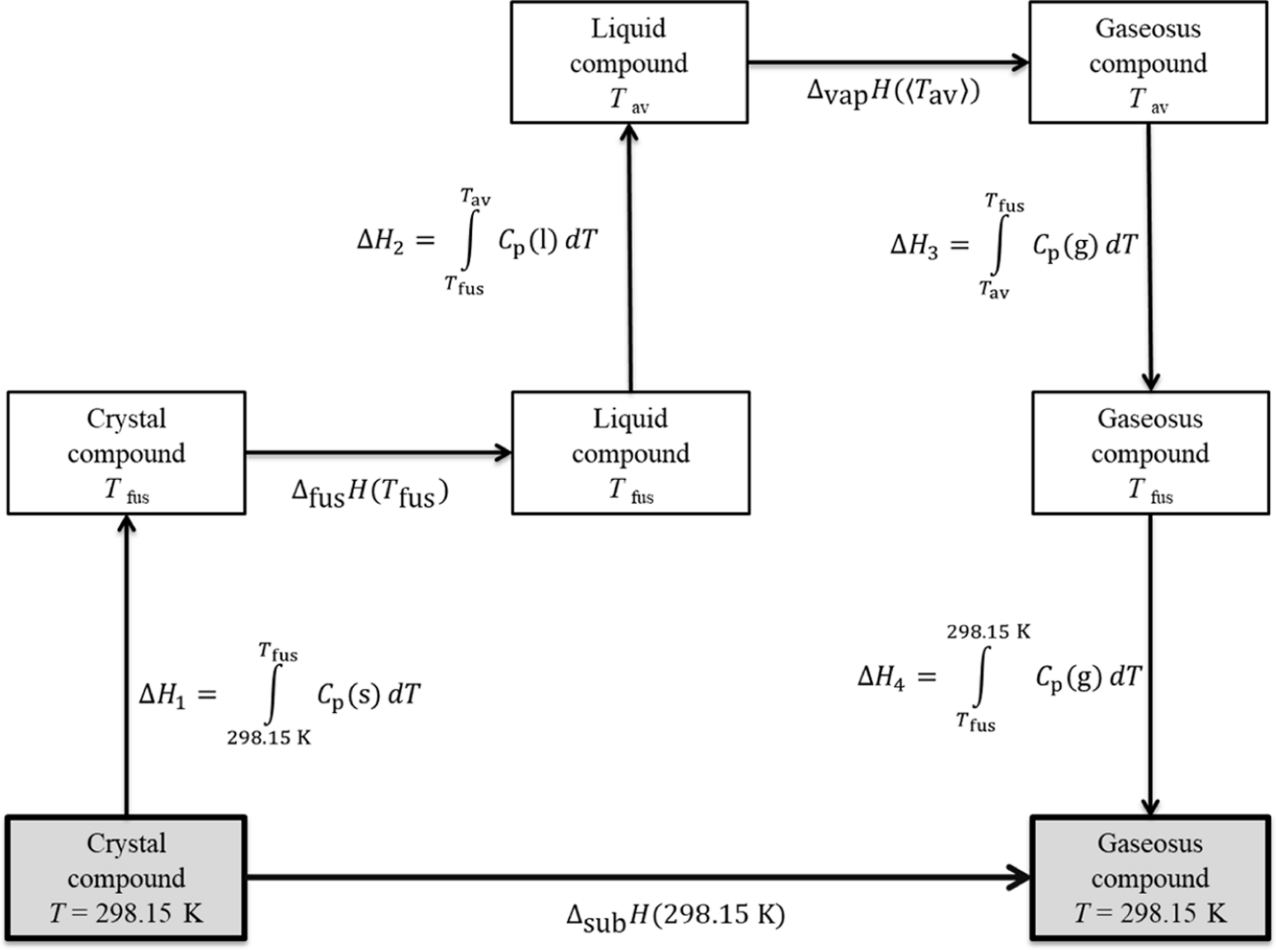
Scheme used to calculate the enthalpies of sublimation at 298.15 K from the enthalpies of vaporization, fusion, and heat capacities of the solid, liquid, and gas phases.

Enthalpies of sublimation at *T* = 298.15 K were calculated from enthalpies of fusion and vaporization as well as from heat capacities of the solid, liquid, and gas phases, as shown in Figure 2; this method has been described previously in ref (12). The diagram depicted in Figure 2 is based on the premise that the enthalpy is a state function and commences considering the solid compound at *T* = 298.15 K, followed by a heating up to the compound’s melting temperature. The enthalpy change of this heating is calculated as . Next, the change from the solid to the liquid phase at *T*_fus_ is considered, which involves the enthalpy of fusion. After the compound is melted, it is further heated from its melting temperature *T*_fus_ to its average vaporization temperature ⟨*T*_vap_⟩; the enthalpy associated with this stage is calculated as . Thereafter, the compound vaporizes, followed by a cooling (in the gas phase) from ⟨*T*_vap_⟩ to *T*_fus_, which implies . Finally, a temperature change occurs from *T*_fus_ to 298.15 K, and the enthalpy of this temperature change is . The Δ*H* terms involving integrals of the heat capacity of the gas phase were solved directly from the partition function *Q* (considering a canonical ensemble; see eq 2, below). The heat capacities of the solid and liquid phases were obtained by DSC (see Table 3). Uncertainties in the enthalpies of sublimation at *T* = 298.15 K are expressed as expanded uncertainties with a coverage factor of *k* = 3.18 and a confidence level of 0.95 (assuming a *t*-student distribution).
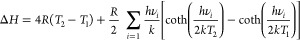
2

## 3 Computational Details

In order to analyze the relation between thermochemical properties and the molecular structure, as well as to provide supporting evidence for the experimental results, molecular orbital calculations were performed. All G4 calculations were carried out using Gaussian09.^34^ NBO analyses^35^ were computed using the program NBO6,^36^ coupled with GAMESS (version 2013-R1).^37^ QTAIM calculations were carried out with DensToolKit.^38^

The formation enthalpies of all compounds were calculated using the atomization method together with the G4 composite method. Enthalpies of formation of gaseous atoms at *T* = 0 K (except for the carbon data, 711.79 kJ·mol^–1^^39^), as well as their thermal corrections at 298.15 K, were taken from the literature.^40^

We used 2DNDPDS^41^ and 4DNDPDS^18^ X-ray crystallography data as starting geometries for geometry optimization calculations. We also considered additional X-ray structures; however, these starting geometries rendered the same final optimized structures (see caption of Table S8 of the Supporting Information for details).

To preserve the consistency among calculations involving vibrational frequencies, the heat capacities were calculated using the vibrational frequencies obtained from the G4 calculation [i.e., at the B3LYP/6-31G(2df,p) theory level], scaled by a factor of 0.9854. This choice is based on the fact that Gaussian09 internally uses this level of theory and scaling factor to compute the G4 energy and enthalpy at 0 K. However, for electron density and NBO analyses, we used the wavefunction (non-relaxed density) of geometries reoptimized at the MP2(full)/cc-pVDZ level of theory. The same level of theory was used to estimate bond dissociation energies of S–S bonds.

## 4 Results and Discussion

### 4.1 Fusion Enthalpies and Heat Capacities

Molar fractions, fusion temperatures, and fusion enthalpies of the disulfides are shown in Table 2. The numerical values of heat capacities of the solid, liquid, and gas phases were adjusted to third-order polynomial equations. The correlation coefficients of the fits are >0.9990, and the adjusted equations are shown in Table 3. The complete data sets of the fusion and heat capacity experiments are shown in Tables S1 and S2 of the Supporting Information.

**Table 2:** Molar Masses, Densities, and Other Properties Determined by DSC of 2DNDPDS and 4DNDPDS at *p*° = 0.1 MPa

compound	chemical formula	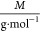 a	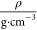	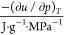	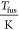 f	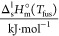 f
2DNDPDS(s)	C_12_H_8_N_2_O_4_S_2_	308.326	1.545^b^	0.200^e^	470.71 ± 0.38	39.45 ± 0.55
4DNDPDS(s)	C_12_H_8_N_2_O_4_S_2_	308.326	1.556^c^	0.200^e^	456.23 ± 0.46	35.81 ± 0.75
benzoic acid(s)	C_7_H_6_O_2_	122.123	1.32^d^	0.116^d^		
cotton(s)	CH_1.742_O_0.921_	28.502	1.50^d^	0.289^d^		

a Molar masses based on IUPAC 2016 recommendation.^19^

b Values taken from ref (17).

c Values taken from ref (18).

d Values taken from ref (42).

e Values taken from ref (24).

f Uncertainties are expressed as expanded uncertainties, calculated from standard uncertainties of Type A. These uncertainties include uncertainties related to calibration, and they were calculated with a coverage factor of *k* = 3.18 and a confidence level of 0.95, considering a *t*-student distribution.^43,44^

**Table 3:** Heat Capacity Equations of the Solid, Liquid, and Gas Phases as a Function of Temperature (*C*_p_(β),β = s, l, g), for 2DNDPDS and 4DNDPDS^a^

compound	temperature range/K	equation	correlation coefficient *r*^2^
2DNDPDS(s, l, g)	283.15–453.15	*C*_p_(s)/J·mol^–1^·K^–1^ – 250.2273 + 3.1898*T*/K – 5.7194 × 10^–3^ (*T*/K)^2^ + 0.4671 × 10^–5^ (*T*/K)^3^	0.9999
	485.15–503.15	*C*_p_(l)/J·mol^–1^·K^–1^ = 1200.2827 × 10^3^ – 7320.4900*T*/K + 14.8834 (*T*/K)^2^–10.0823 × 10^–3^ (*T*/K)^3^	0.9965
	298.15–500.00	*C*_p_(g)/J·mol^–1^·K^–1^ = 7.0475 + 1.0326*T*/K – 3.6314 × 10^–4^ (*T*/K)^2^ – 2.2867 × 10^–7^·(*T*/K)^3^	1.0000
4DNDPDS(s, l, g)	288.15–423.15	*C*_p_(s)/J·mol^–1^·K^–1^ = 775.9189 – 4.1127*T*/K + 10.2484 × 10^–3^ (*T*/K)^2^ – 0.5926 × 10^–5^ (*T*/K)^3^	0.9993
	473.15–506.15	*C*_p_(l)/J·mol^–1^·K^–1^ = 71679.9206 – 461.1313*T*/K + 989.9387 × 10^–3^ (*T*/K)^2^ + 70.4097 × 10^–5^ (*T*/K)^3^	0.9998
	298.15–500.00	*C*_p_(g)/J·mol^–1^·K^–1^ = 9.7533 + 1.0230*T*/K – 3.5072 × 10^–4^ (*T*/K)^2^ – 2.3512 × 10–^7^ (*T*/K)^3^	1.0000

a *C*_*p*_(s) and *C*_*p*_(l) were determined by DSC, and *C*_*p*_(g) was computed using molecular orbital calculations. Standard uncertainty *u*(*T*) = 0.01 K. Regarding properties of the solid and liquid phases, the uncertainties were calculated from standard uncertainties of Type A, and they include contributions from calibration: *U*(*C*_p_, 2DNDPDS, s) = 6.0 J·mol^–1^·K^–1^, *U*(*C*_p_, 2DNDPDS, l) = 7.0 J·mol^–1^·K^–1^, *U*(*C*_p_, 4DNDPDS, s) = 4.6 J·mol^–1^·K^–1^, and *U*(*C*_p_, 4DNDPDS, l) = 9.0 J·mol^–1^·K^–1^; the uncertainties are expressed at a level of confidence of 0.95 and a coverage factor *k* = 2.78, considering a *t*-student distribution.

Some selected melting temperatures of 2DNDPDS and 4DNDPDS, as have been reported in the literature, are shown in Table 4. The temperatures are in good agreement with our results, although the data reported in other works were obtained through inaccurate techniques. To the best of our knowledge, there are no previous reports where to find fusion enthalpies or heat capacities of 2DNDPDS and 4DNDPDS.

**Table 4:** Fusion Temperatures Reported in the Literature and As-Obtained in the Present Work of 2DNDPDS and 4DNDPDS

refs	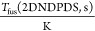	refs	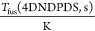
(45)	468.15–470.15	(48)	456.15
(46)	468.15	(49)	457.15–458.15
(47)	470.15	(50)	455.15–457.15
this work	470.71 ± 0.38^a^	this work	456.23 ± 0.46^a^

a The numerical values that follow the “±” symbol are expanded uncertainties, which include the contributions of the calibration, and are expressed at a coverage factor of *k* = 3.18 and a confidence level of 0.95, assuming a *t*-student distribution.

### 4.2 Combustion Energies and Enthalpies

Table 5 shows the specific combustion energies of 2DNDPDS and 4DNDPDS. Each numerical value is the average of seven experiments, and uncertainties are expressed as standard uncertainties. The details of every combustion experiment are provided in Tables S4 and S5 of the Supporting Information. The idealized combustion reaction presented in eq 3 was used in our procedures.
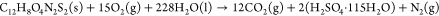
3

**Table 5:** Individual Values of the Mass Energy of Combustion of 2DNDPDS and 4DNDPDS at *T* = 298.15 K and *p*° = 0.1 MPa^a^

2DNDPDS(s)	4DNDPDS(s)
(−Δ_c_*u*°)/J·g^–1^	
23122.3	23056.1
23112.0	23030.8
23130.7	23096.6
23149.5	23067.2
23112.2	23064.2
23.132.8	23031.0
23137.3	23074.1
⟨−Δ_c_u°/J·g^–1^⟩	
23128.1 ± 5.2	23060.0 ± 8.9

a Uncertainties are expressed as standard uncertainties.

Molar standard energies of combustion [Δ_c_*U*°(s)], standard molar enthalpies of combustion [Δ_c_*H*°(s)], and standard molar enthalpies of formation in the solid phase [Δ_f_*H*°(s)], for 2DNDPDS and 4DNDPDS at 298.15 K, are presented in Table 6.

**Table 6:** Standard Molar Energies and Enthalpies of Combustion and Formation of 2DNDPDS and 4DNDPDS at *T* = 298.15 K and *p*° = 0.1 MPa^a^

compound	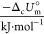	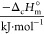	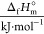
2DNDPDS(s)	7131.2 ± 2.3	7136.1 ± 2.3	66.7 ± 2.7
4DNDPDS(s)	7110.2 ± 3.2	7115.1 ± 3.2	45.7 ± 3.5

a Uncertainties expressed as expanded uncertainties of Type A, which include contributions from calibration, and from uncertainties in combustion energies of benzoic acid, auxiliary materials, and the compounds studied. In addition, they were calculated with a coverage factor of *k* = 1.96 so as to render a confidence level of 0.95.

### 4.3 Vaporization and Sublimation Enthalpies

Some preliminary tests showed that the TA Instruments Q500 could not detect the mass losses occurring before the melting temperature of each disulfide. In contrast, the mass loss was properly quantified above that temperature when the sample was liquid. Therefore, we studied vaporization over the temperature ranges (480–500) K and (470–490) K for 2DNDPDS and 4DNDPDS, respectively. We determined the temperature dependency of the mass loss rate from thermogravimetric curves. Subsequently, we tabulated ln (d*m*/d*t*·*T*) and 1/*T*, and fitted this to eq 1. The respective slope was used to determine the enthalpy of vaporization at the mean temperature (average over the range analyzed) ⟨*T*_vap_⟩. A typical data set of a 2DNDPDS thermogravimetric experiment is shown in Table 7, together with the respective correlation coefficient *r*^2^, and the uncertainties in the intersection σ_a_ and in the slope σ_b_. The thermogravimetric experiments are shown in detail in Tables S6 and S7 of the Supporting Information. All data necessary to derive sublimation enthalpies at *T* = 298.15 K according Figure 2 are presented in Table 8.

**Table 7:** Thermogravimetric Data of a Representative Experimental Series of 2DNDPDS and the Weighted-Average Vaporization Enthalpies of 2DNDPDS and 4DNDPDS^a^

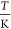	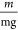	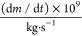	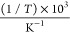	ln(d*m*/d*t*·*T*)
2DNDPDS(l) series 1				
480.0	25.7697	1.4869	2.083	–14.153
482.0	25.7497	1.6239	2.075	–14.060
484.0	25.7278	1.7803	2.066	–13.964
486.0	25.7042	1.9581	2.058	–13.865
488.0	25.6792	2.1276	2.049	–13.778
490.0	25.6521	2.3373	2.041	–13.680
492.0	25.6226	2.5593	2.033	–13.585
494.0	25.5907	2.7791	2.024	–13.499
496.0	25.5556	3.0463	2.016	–13.403
498.0	25.5175	3.3102	2.008	–13.316
500.0	25.4760	3.6073	2.000	–13.226
series 1 ln(d*m*/d*t*·*T*) = 9.1–11156.4/*T*; *r*^2^ = 0.9999; σ_a_ = 0.1; σ_b_ = 28.9; Δ_l_^g^*H*_m_(490.0 K)/kJ·mol^–^^1^= 92.8 ± 0.2				
series 2 ln(d*m*/d*t*·*T*) = 8.9–11105.3/*T*; *r*^2^ = 0.9971; σ_a_ = 0.4; σ_b_ = 200.8; Δ_l_^g^*H*_m_(490.0 K)/kJ·mol^–^^1^= 90.3 ± 1.7				
series 3 ln(d*m*/d*t*·*T*) = 8.7–11012.1/*T*; *r*^2^ = 0.9970; σ_a_ = 0.4; σ_b_ = 201.4; Δ_l_^g^*H*_m_(490.0 K)/kJ·mol^–^^1^= 91.6 ± 1.7				
series 4 ln(d*m*/d*t*·*T*) = 9.2–11197.8/*T*; *r*^2^ = 0.9998; σ_a_ = 0.1; σ_b_ = 56.3; Δ_l_^g^*H*_m_(490.0 K)/kJ·mol^–^^1^= 93.1 ± 0.5				
⟨Δ_l_^g^*H*_m_(2DNDPDS, 490.0 K)⟩/kJ·mol^–1^ = 92.8 ± 0.4				
4DNDPDS(l)				
series 1 ln(d*m*/d*t*·*T*) = 9.9–11798.7/*T*; *r*^2^ = 0.9974; σ_a_ = 0.4; σ_b_ = 200.3; Δ_l_^g^*H*_m_(480.0 K)/kJ·mol^–^^1^= 98.1 ± 1.7				
series 2 ln(d*m*/d*t*·*T*) = 10.4–12134.2/*T*; *r*^2^ = 0.9992; σ_a_ = 0.2; σ_b_ = 112.9; Δ_l_^g^*H*_m_(480.0 K)/kJ·mol^–^^1^= 100.9 ± 0.9				
series 3 ln(d*m*/d*t*·*T*) = 9.7–11797.5/*T*; *r*^2^ = 0.9956; σ_a_ = 0.5; σ_b_ = 261.0; Δ_l_^g^*H*_m_(480.0 K)/kJ·mol^–^^1^= 98.1 ± 2.2				
series 4 ln(d*m*/d*t*·*T*) = 10.5–12211.0/*T*; *r*^2^ = 0.9970; σ_a_ = 0.5; σ_b_ = 224.7; Δ_l_^g^*H*_m_(480.0 K)/kJ·mol^–^^1^= 101.5 ± 1.9				
⟨Δ_l_^g^*H*_m_(4DNDPDS, 480.0 K)⟩/kJ·mol^–1^ = 100.2 ± 1.4				

a Standard uncertainties *u* are *u*(*T*) = 0.1 K and *u*(*m*) = 0.1 μg, and the combined expanded uncertainties *U*_c_ are *U*_c_(d*m*/d*t*) = 0.066 × 10^9^ kg·s^–1^, *U*_c_(1/*T*) = 0.001 × 10^3^ K^–1^, *U*_c_(ln(d*m*/d*t*·*T*^1/2^)) = 0.020, and *U*_c_(ln(d*m*/d*t*·*T*)) = 0.020 (0.95 level of confidence).

**Table 8:** Experimental Values of Fusion and Vaporization Enthalpies, and Temperature Corrections Required to Calculate the Sublimation Enthalpies at *T* = 298.15 K for the Diphenyl Disulfides Studied Here

compound	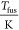 a	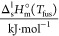 a	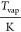 b	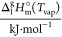 a	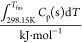 c	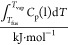 c	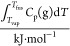	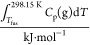	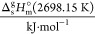 d
2DNDPDS(s,l)	470.71 ± 0.38	39.45 ± 0.55	490.0	92.8 ± 1.3	68.2 ± 0.1	10.6 ± 0.1	–7.6	–57.9	145.6 ± 1.4
4DNDPDS(s,l)	456.23 ± 0.46	35.81 ± 0.75	480.0	100.2 ± 2.7	59.0 ± 0.3	12.1 ± 0.3	–9.2	–52.5	145.4 ± 2.8

a Uncertainties are expressed as expanded uncertainties, which include the contributions stemming from the calibration, and calculated with a coverage factor of *k* = 3.18 and a confidence level of 0.95, considering a *t*-student distribution.

b Standard uncertainties *u*(*T*) = 0.1 K.

c Numbers following the “±” symbol are expanded uncertainties, each of which includes the contributions originated from the uncertainty in the heat capacity of the solid or liquid phases, with a coverage factor *k* = 2.78 and a confidence level of 0.95 and assuming a *t*-student distribution.

d Uncertainty calculated as the square root of the sum of squared uncertainties in the terms described in the pathway of Figure 2.

### 4.4 Enthalpies of Formation in the Gas Phase

Each standard molar formation enthalpy in the gas phase (Δ_f_*H*_m_^°^(g)) was calculated by adding the respective sublimation enthalpy (Δ_s_^g^*H*_m_^°^) and formation enthalpy in the crystalline phase (Δ_f_*H*_m_^°^(s)), both at *T* = 298.15 K. The uncertainties in gas-phase formation enthalpies were calculated as the square root of the sum of squared uncertainties of formation enthalpies in the crystalline phase and of sublimation enthalpies. Experimental and theoretical Δ_f_*H*_m_^°^(g)’s are compared in Table 9.

**Table 9:** Standard Molar Enthalpies of Formation of 2DNDPDS and 4DNDPDS at *T* = 298.15 K

compound	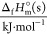 a	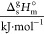 b	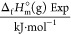 c	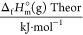 d
2DNDPDS(s)	66.7 ± 2.7	145.6 ± 1.4	212.3 ± 3.0	207.1 (5.2)
4DNDPDS(s)	45.7 ± 3.5	145.4 ± 2.8	191.1 ± 4.5	189.9 (1.2)

a The number following the “±” is the expanded uncertainty, which includes the uncertainties stemming from the calibration, and the combustion energies of benzoic acid, auxiliary materials, and the respective disulfide with a coverage factor *k* = 1.96 and a confidence level of 0.95.

b Numbers following the “±” symbol are expanded uncertainties, which include contributions from uncertainties in the heat capacity in the solid or liquid phases with a coverage factor *k* = 3.18 and a confidence level of 0.95.

c The number following the “±” symbol is the uncertainty calculated by applying the root of the sum of squares method.

d The number enclosed in parentheses is , in kJ·mol^–1^.

**Figure 3:**
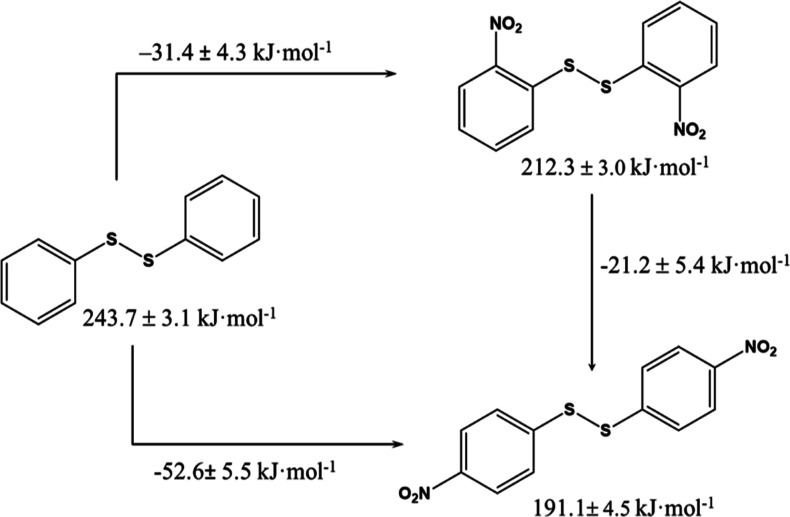
Enthalpic contribution of inserting a nitro group to diphenyl disulfide at positions 2 and 4 (horizontal rows), and isomerization enthalpy from 2DNDPDS to 4DNDPDS (vertical row).

**Figure 4:**
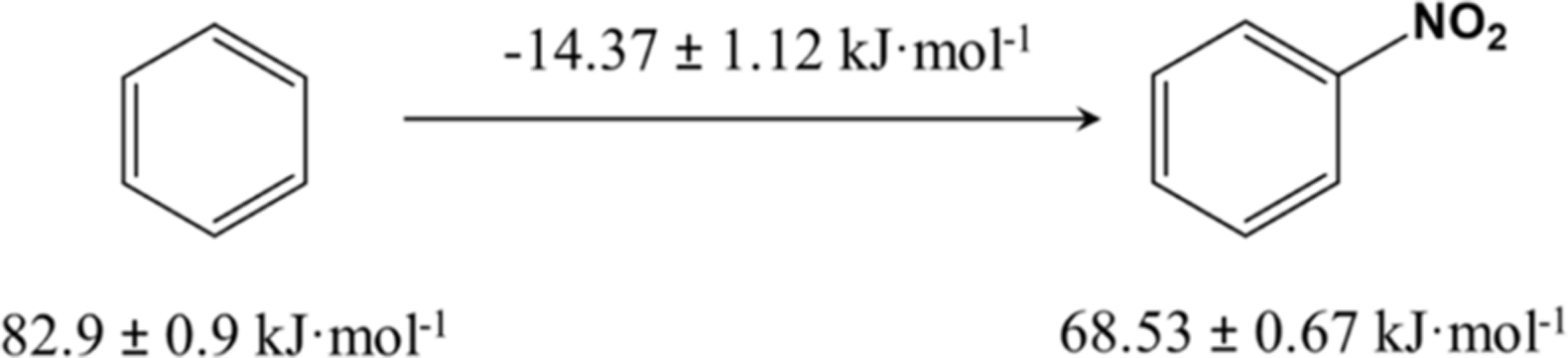
Enthalpic contribution (in the gas phase) due to the insertion of a nitro group into benzene.

From the enthalpies of formation in the gas phase, enthalpy increases were obtained (Figure 3) by exchanging the (−H) and (−NO_2_) groups at the corresponding positions of diphenyl disulfide. The gas formation enthalpy of diphenyl disulfide is 243.7 ± 3.1 kJ·mol^–1^, which was reported previously.^12^ According to Figure 3, inserting two (−NO_2_) groups at positions 2 and 4, respectively, has an exothermic effect of (−31.4 ± 4.3) kJ·mol^–1^ and (−52.6 ± 5.5) kJ·mol^–1^. On the other hand, if we consider that the previous enthalpic increase can be decomposed into two independent (−H)/(−NO_2_) exchanges, then inserting one (−NO_2_) group at positions 2 and 4 of the diphenyl disulfide causes an enthalpy change of (−15.7 ± 4.3) kJ·mol^–1^ and (−26.3 ± 5.5) kJ·mol^–1^, respectively. This increase is close to the enthalpy increase caused by inserting a (−NO_2_) group into benzene to produce nitrobenzene (Figure 4). Let us recall that the enthalpies of formation of benzene and nitrobenzene in the gas phase are (82.9 ± 0.9) kJ·mol^–1^^51^ and (68.53 ± 0.67) kJ·mol^–1^,^52^ respectively; hence the associated enthalpy increase is (−14.37 ± 1.12) kJ·mol ^–1^. In addition, we observe that moving the (−NO_2_) group from position 2 to 4 (i.e., isomerizing 2DNDPDS to 4DNDPDS) implies an isomerization enthalpy of (−21.2 ± 5.4) kJ·mol^–1^. As we will discuss in the following sections, the overall stabilization gain may be attributed to the combined contributions of the blue shifting of the C–H···S hydrogen bonds, the increased resonance between the (−NO_2_) and the aryl ring (favored from an almost perfect coplanarity of these groups), both present in 4DNDPDS, and the strengthening of the S–S bond in 4DNDPDS.

### 4.5 Gas Phase Molecular Structures

#### 4.5.1 Geometric Considerations

**Figure 5:**
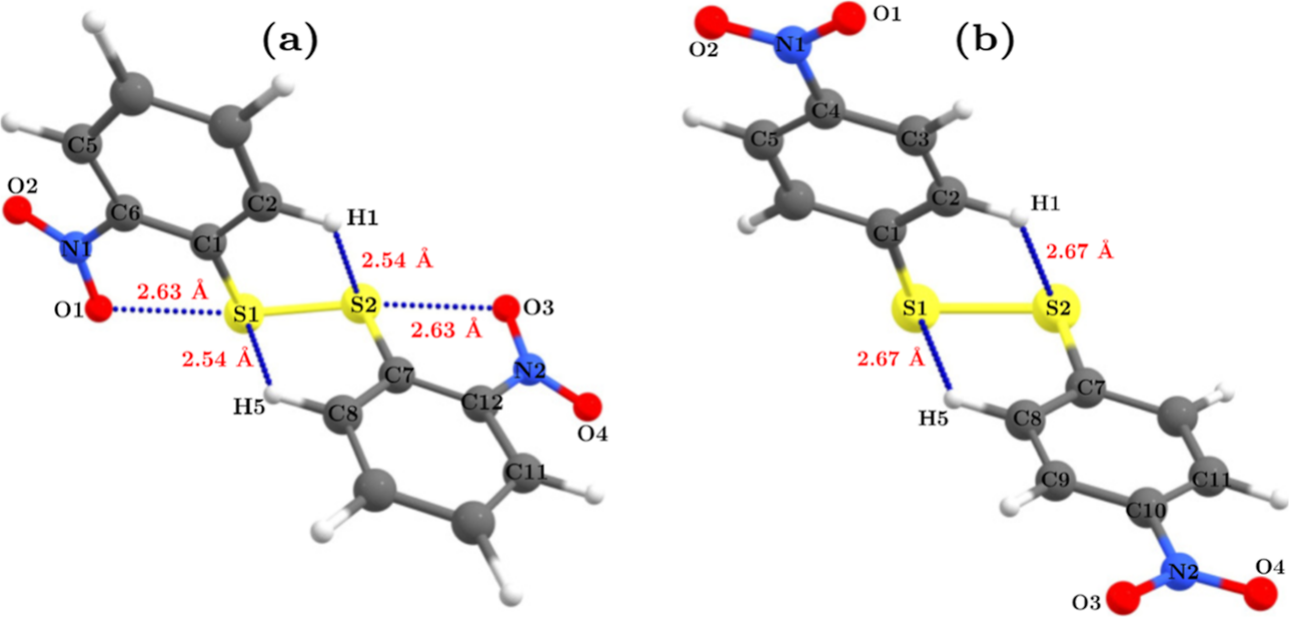
Optimized structures of (a) 2DNDPDS and (b) 4DNDPDS at the MP2(full)/cc-pVDZ level of theory.

In Figure 5, we show the optimized molecular structures of 2DNDPDS and 4DNDPDS, and in Table 10, we list the geometric parameters that will be relevant to our discussion. In both disulfides, we observe a C_2_ symmetry; therefore, unless stated otherwise, we will perform our discussion considering only half the respective disulfide, taking for granted that the same observations applied to its other half. For the interested reader, in Table S8 of the Supporting Information, we also provide additional geometric parameters obtained from additional X-ray structures.

**Table 10:** Relevant Geometric Parameters and Bond Dissociation Energies of the Optimized Structures of 2DNDPDS and 4DNDPDS at the MP2(full)/Cc-pVDZ Level of Theory^a^

	structures	
parameters	2DNDPDS^b^	4NFPDS^c^
S1–S2 (Å)	*2.090(2.045)*	*2.065(2.019)*
C1–S1–S2–C7 (°)	*86.4(85.1)*	*84.6(90.1)*
O1···S1–S2 (°)	*172.6(173.5)*	
O3···S2–S1 (°)	*172.6(167.2)*	
O1···S1 (Å)	*2.631(2.588)*	
O3···S2 (Å)	*2.631(2.636)*	
S1–C1 (Å)	*1.799(1.785)*	*1.790(1.767)*
S2–S1–C1 (°)	*102.4(104.2)*	*104.0(106.2)*
S2–S1–C1–C2 (°)	*–15.6(−19.1)*	*–15.7(−21.7)*
S2–C7 (Å)	*1.799(1.808)*	*1.790(1.767)*
S2–S1–C1 (°)	*102.4(104.2)*	*104.0(106.2)*
S2–S1–C1–C2 (°)	*–15.6(−19.1)*	*–15.7(−21.7)*
N1–C6 (Å)	*1.471(1.476)*	
N2–C12 (Å)	*1.471(1.425)*	
τ_1_ (°)	*19.0(17.8)*	
N1–C4 (Å)		*1.478(1.488)*
N2–C10 (Å)		*1.478(1.488)*
τ_2_ (°)		*0.2(8.2)*
H1···S2 (Å)	*2.536(2.570)*	*2.671(2.795)*
H5···S1 (Å)	*2.536(2.639)*	*2.671(2.795)*
C2–H1···S2 (°)	*114.2(118.4)*	*110.7(111.6)*
C8–H5···S1 (°)	*114.2(114.5)*	*110.7(111.6)*
BDE(S1–S2) (kJ·mol^–1^)	*239.15*	*280.65*

a τ_1_ = (ω_1_ + ω_2_)/2, τ_2_ = (ω_3_ + ω_4_)/2; ω_1_ = O1(3)–N1(2)–C6(12)–C1(7), ω_2_ = O2(4)–N1(2)–C6(12)–C5(11), ω_3_ = O1(3)–N1(2)–C4(10)–C3(9), ω_4_ = O2(4)–N1(2)–C4(10)–C5(11).

b Values in italics were taken from ref (41).

c Values in italics were taken from ref (18).

A notable feature of molecules that contain an −NO_2_ group bonded to an aromatic ring is the possible coplanarity between the latter groups. This coplanarity or its deviation is the result of electronic and steric effects between the −NO_2_ and the aromatic ring. We analyze this feature quantitatively by averaging the dihedral angles ω_1_ = O1–N1–C6–C1 and ω_2_ = O2–N1–C6–C5, for 2DNDPDS, and the dihedral angles ω_3_ ≡ O1–N1–C4–C3 and ω_4_ ≡ O2–N1–C4–C5, for 4DNDPDS (see Figure 3 for the atom numbering). The averages are defined and denoted as follows: τ_1_ ≡ (ω_1_ + ω_2_)/2 and τ_2_ ≡ (ω_3_ + ω_4_)/2, for 2DNDPDS and 4DNDPDS, respectively.

In 4DNDPDS, the −NO_2_ and the aromatic ring groups are coplanar (τ_2_ = 0.2°), which stems from the electronic delocalization between these groups. In contrast, in the minimum energy conformation of 2DNDPDS, the same groups show a nonzero torsion angle τ_1_ = 19.0° relative to the plane that contains the aryl ring. Here, the non-coplanarity, which might be counterintuitive, allows the formation of attractive interactions between oxygen and sulfur atoms. The attractive character of these interactions can be inferred from the distances O1–S1 and S2–O3 (2.631 Å), which are much smaller than the sum of their van der Waals radii (3.25 Å). The shortening of the distances stems from the alignment of O1···S1–S2···O3 (the angles O1···S1–S2 and S1–S2···O3 are almost 180°), which in turn allows the formation of an *n*_(O)_ → σ_(S–S)_* ← *n*_(O)_ hypervalent four-center six-electron bond. This kind of interaction has been studied previously in bis[(8-phenylthiol)naphthyl]disulfide for a four-sulfur-atom bond (S···S–S···S),^53^ and in bis[(2-(1-*H*-benzimidazol-2 −yl)phenyl]disulfide for an N···S–S···N interaction.^54^

Furthermore, we also observe that the S–S distance in 2DNDPDS (2.090 Å) is slightly greater than in 4DNDPDS (2.065 Å). This stretch suggests that the formation of the *n*_(O)_ → σ_(S–S)_* ← *n*_(O)_ interaction weakens the S–S bond, relative to the 4DNDPDS isomer. Quantitatively, we computed the bond dissociation energies (BDE) of the S–S bonds, and indeed, the BDE is 41.50 kJ·mol^–1^ greater for 4DNDPDS (see Table 10).

Another exciting geometric feature found in both structures is the presence of two C–H···S contacts. In Table 10, see the distances H4···S2 in 2DNDPDS (2.536 Å) and H1···S2 in 4DNDPDS (2.671 Å); they are smaller than the sum of the van de Waals radii of H and S (3.03 Å). The observed H···S distances are also considerably smaller than the most-frequently-observed H···S distance occurring in the solid state (3.21 Å).^55^ In addition, the C–H···S angles are 114.2 and 107.7° in 2DNDPDS and 4DNDPDS, respectively. All these parameters suggest the presence of C–H···S intramolecular hydrogen bonds in both structures. While C–H···S interactions are seldom reported, their relevance has been accepted,^53,56^ and it will be interesting to analyze their influence on the minimum energy structures of 2DNDPDS and 4DNDPDS, as we will show in the next section.^57^

#### 4.5.2 NBO Analysis

##### 4.5.2.1 –NO_2_ Group as an Electron Acceptor Moiety

In order to assess the effect of the −NO_2_ group as an electron density acceptor, we apply the natural bond orbital (NBO) analysis to the bonds N1–O1 and N1–O2 for 2DNDPDS and to the bonds C3–C4, C4–C5, N1–O1, and N1–O2 for 4DNDPDS.

**Figure 6:**
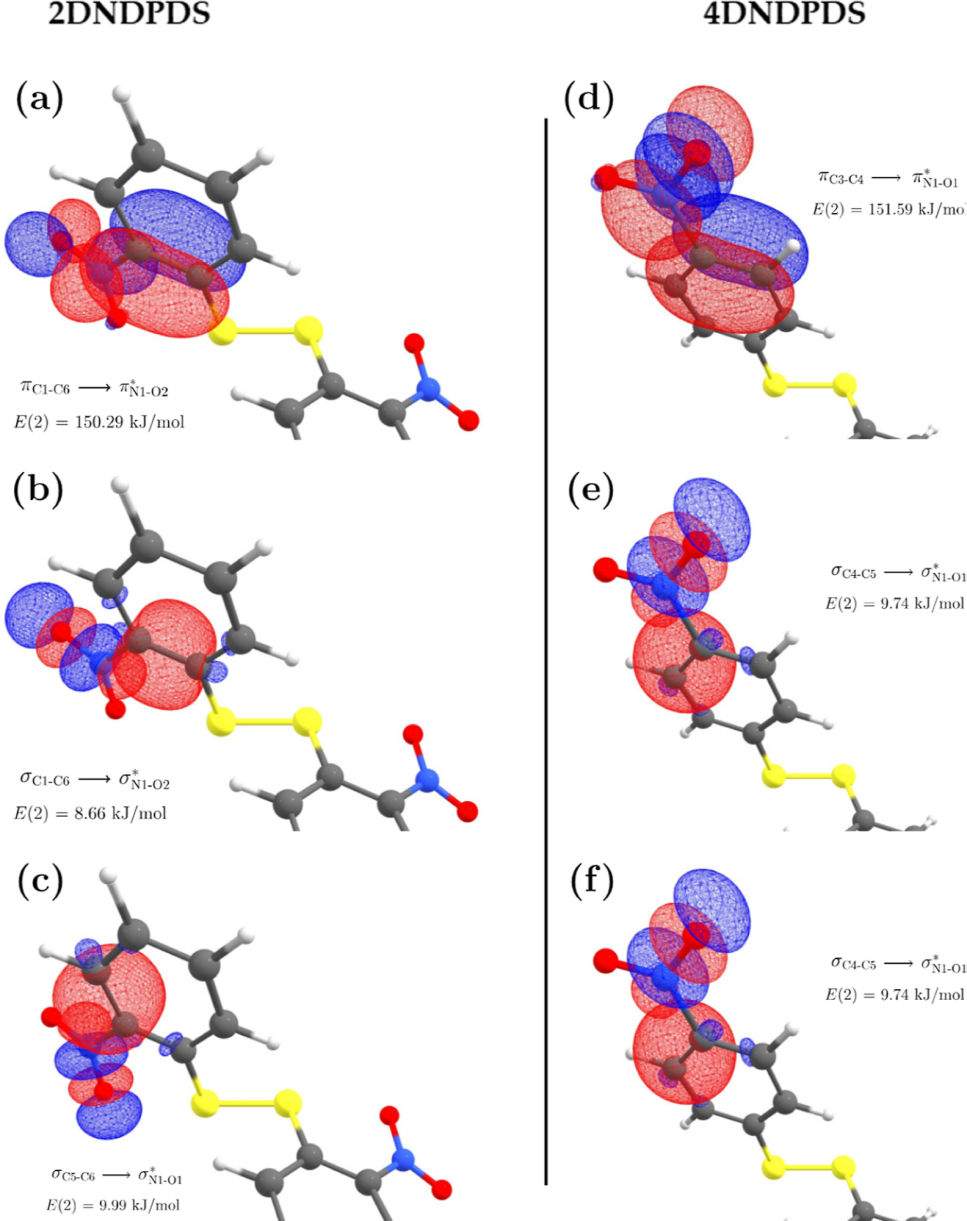
Electron acceptor character of the −NO_2_ group (i.e.*,* the σ → σ* and π → π* interactions) in 2DNDPDS (left column) and in 4DNDPDS (right column), respectively. In each subfigure, only half the molecule is shown.

The electron acceptor orbital interactions involving the −NO_2_ group are of the kind π → π* and σ → σ*. Figure 6 shows the involved NBOs and their respective stabilization energies, *E*(2), for both disulfides. The total stabilization energies of these orbitals, *E*_Σ,ac_(2), are 168.94 and 171.07 kJ·mol^–1^ for 2DNDPDS and 4DNDPDS, respectively, which implies that the −NO_2_ groups are slightly more stable in 4DNDPDS. However, it is remarkable that even when the −NO_2_ group shows a nonzero torsion angle relative to the aromatic ring (τ_1_ = 19°, see Table 10), the group still has a high stabilization energy due to electron acceptor orbital interactions.

##### 4.5.2.2 Attractive *n*_(O)_ → σ_(S–S)_* ← *n*_(O)_ Versus Repulsive *n*_(O)_)(*n*_(S)_ Interactions

**Figure 7:**
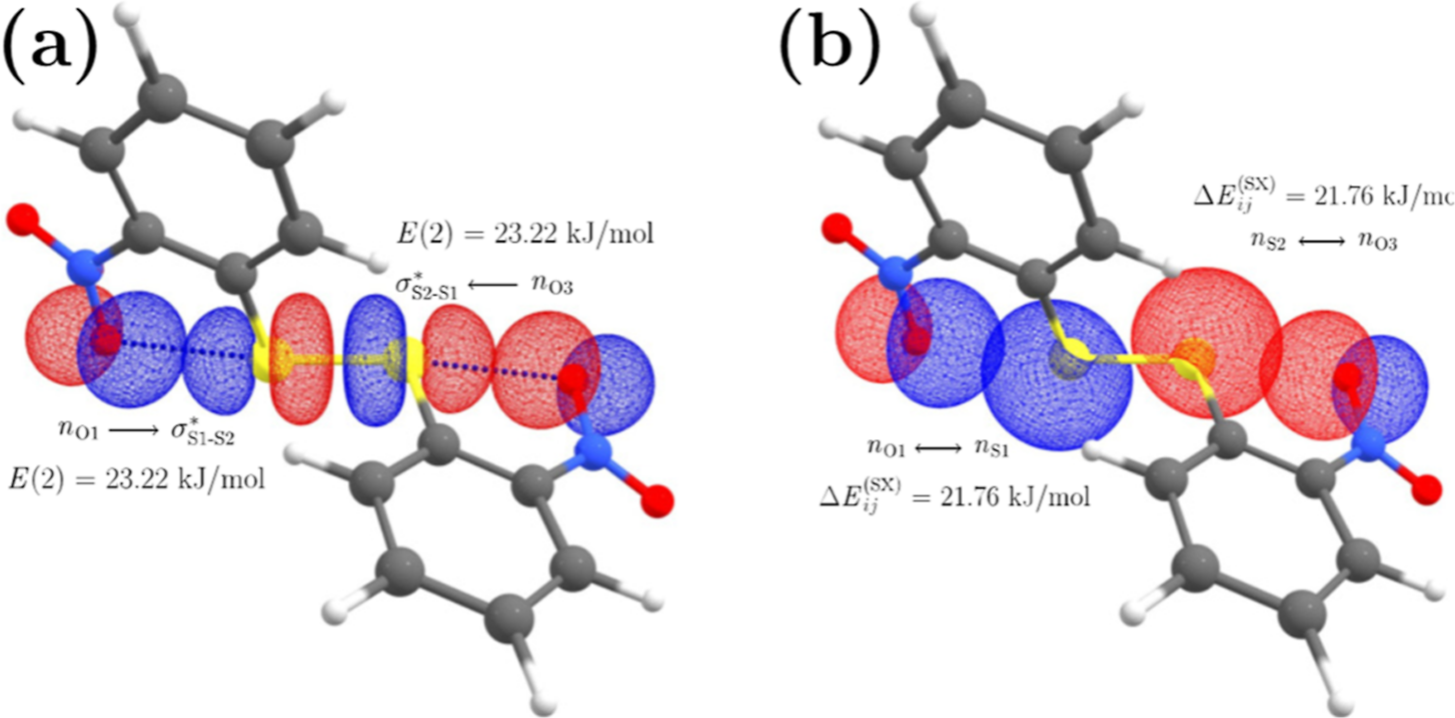
NBO interactions in 2DNDPDS: (a) *n*_O1_ → σ_S1–S2_ ← *n*_O3_ and (b) *n*_O1(3)_ ↔ *n*_S1(2)_.

As mentioned above, the geometric parameters suggest that a hypervalent four-center six-electron (4c–6e) interaction *n*_(O)_ → σ_(S–S)_* ← *n*_(O)_ is present in 2DNDPDS, even when there is an intuitively expected steric repulsion between O1(3) ↔ S1(2) atoms. In what follows, we analyze these interactions using NBO and NBO STERIC.^35^ In Figure 7a, we show the overlap of NBOs that favors the alignment of O1···S1–S2···O3 atoms in 2DNDPDS. The energy, *E*(2), of the interaction *n*_(O1)_ → σ_(S1–S2)_* ← *n*_(O3)_ adds to 46.44 kJ·mol^–1^. On the other hand, the repulsive steric effect of the *n*_(O)_)(*n*_(S)_ interaction was estimated through the steric exchange energy, Δ*E*_*j*_^(sx)^, between the filled localized NBOs *n*_(O1)_ ↔ *n*_(S1)_ and *n*_(S2)_ ↔ *n*_(O3)_, whose total exchange energy is 43.52 kJ·mol^–1^. Interestingly, the hypervalent 4c–6e interaction compensates the instability stemming from the steric repulsion by 2.92 kJ·mol^–1^. Furthermore, even though the energy *E*(2) of a single interaction *n*_(O)_ → *σ*_(S)_* is small (23.22 kJ·mol^–1^), approximately the order of a weak hydrogen bond energy, it is enough to influence the molecular structure, i.e., the hypervalent interaction compensates the steric effects and the non-planarity between the −NO_2_ groups and the neighbor aromatic rings.

##### 4.5.2.3 Intramolecular Weak Hydrogen Bond C–H···S

**Figure 8:**
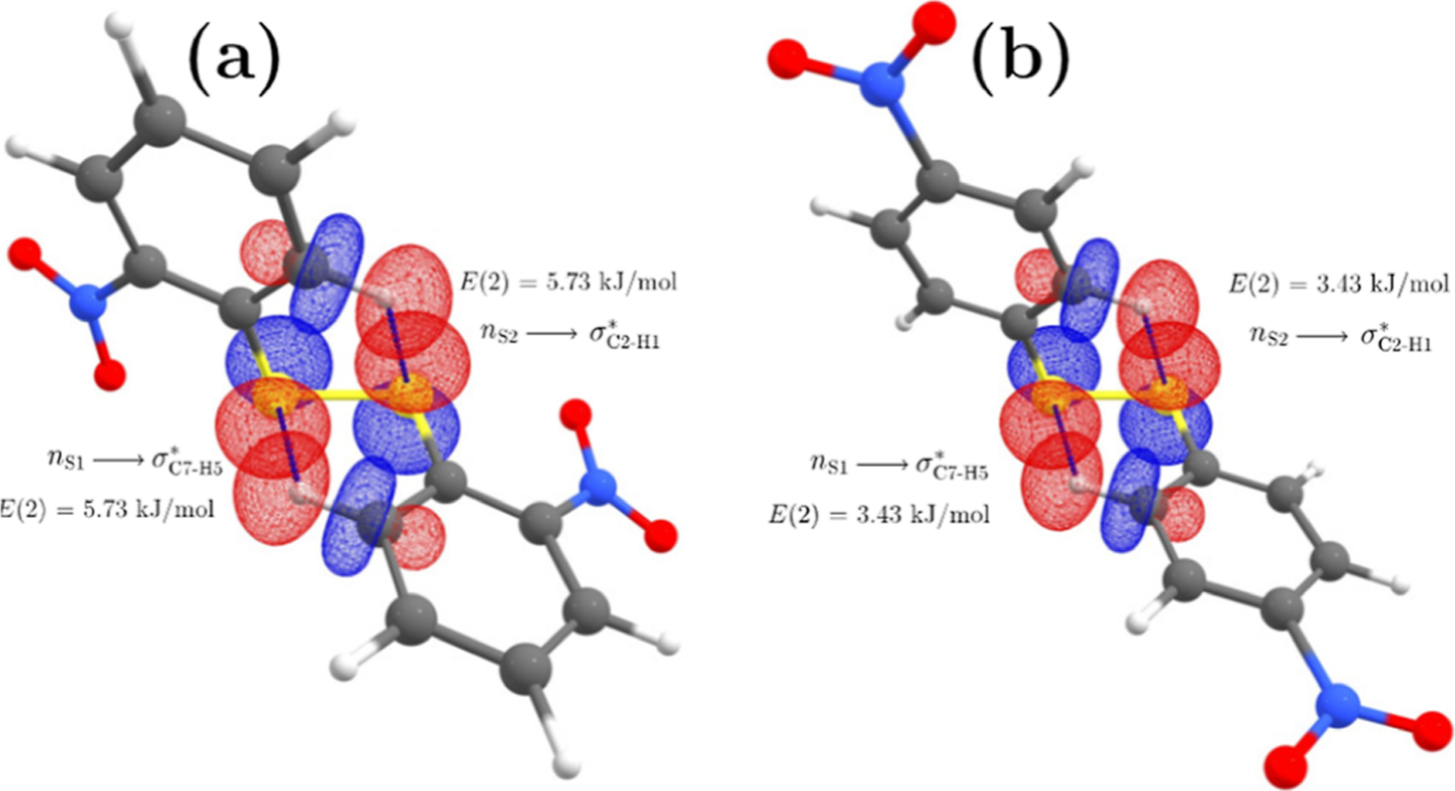
C–H···S hydrogen bonds present in (a) 2DNDPDS and (b) 4DNDPDS.

C–H···S hydrogen bonds are classified as nonclassical (*aka* “blue shifting”) hydrogen bonds.^55,58−60^ The blue shifting of the stretching frequency of a nonclassical C–H hydrogen bond stems from the C atom rehybridization and the resulting C–H bond polarization.^61,62^ In the disulfides studied here, the NBO analysis reveals that the hyperconjugation interactions *n*_(S)_ → σ_(C_–_H)_* in 2DNDPDS and 4DNDPDS might be considered weak, as their stabilization energies *E*(2) are <6 kJ·mol^–1^ (see Figure 8). However, we notice the two following significant changes to the C2–H1 and C7–H5 bonds. (1) The s character of hydrogen donor atoms increases to 29.33 and 29.50% for 2DNDPDS and 4DNDPDS, versus the expected 25% of an ideal sp^3^ hybrid orbital. (2) The C2–H1 (and C8–H5) bond polarization is 62.71% for C and 37.29% for H in 2DNDPDS, and 62.62% for C and 37.38% for H in 4DNDPDS, as opposed to 50/50% of a non-polarized C–H bond. These s-characters and polarizations are larger, relative to the polarizations found for other C–H bonds, which are not of the type C–H···S (e.g., consider the C6–H4 bond in 4DNDPDS: the C has an s character of 28.87% and its polarization is 62.18% for C and 37.82% for H).

From the previous results, it is observed that the C–H···S bond in 4DNDPDS has a slightly larger “blue shifting” character than in 2DNDPDS. The above results also suggest that the rehybridization of C2 (C8) and the polarization of the C2–H1 (C8–H5) bond are the dominant effects constituting the hyperconjugation *n*_(S1)_ → σ_(C2_–_H1)_* (*n*_(S2)_ → σ_(C8_–_H5)_*), which is consistent with previous work.^61,62^

#### 4.5.3 QTAIM Analysis

**Figure 9:**
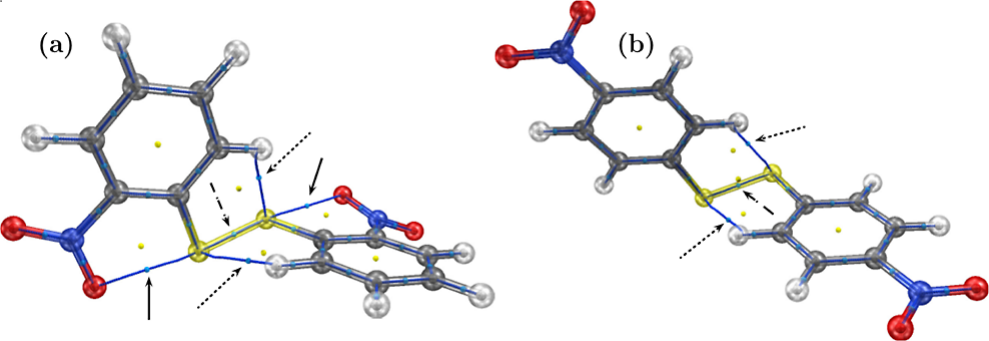
Molecular graphs of (a) 2DNDPDS and (b) 4DNDPDS. Solid arrows point to bond critical points (BCPs) of weak O···S interactions, dashed arrows point to BCPs of S–S bonds, and dotted arrows point to BCPs of weak S···H interactions.

In this section, we use Bader’s quantum theory of atoms in molecules (QTAIM),^63^ in order to provide further evidence on the formation of hypervalent 4c–6e bonding in 2DNDPDS as well as to analyze C–H···S bonds in more depth. For the QTAIM analysis, we used wavefunctions computed at the MP2(full)/cc-pVDZ theory level. Figure 9 depicts the molecular graphs of both disulfides. In Figure 9a, the solid arrows point to bond critical points (BCPs) of 2DNDPDS, which indicate weak interactions between O1···S1 and O3···S2; together with the BCP between S1 and S2 (pointed to by dashed arrows in Figure 6a), the existence of a hypervalent 6e–4c interaction O1···S1–S2···O3 is confirmed. Furthermore, the electron density (ρ) and its Laplacian (∇^2^ρ) at the BPCs of both O···S contacts are 0.02483 and 0.08412 a.u., respectively; these values are within the typical range for interactions of this kind.^64^

In addition, in both disulfides, we found BCPs and gradient bond paths connecting S1···H1 and S2···H5. ρ and ∇^2^ρ at the respective BCPs are 0.0173 and 0.0543 a.u. for 2DNDPDS, and 0.0137 and 0.0450 a.u. for 4DNDPDS. These values are within the typical range (i.e., 0.002 ≤ ρ ≤ 0.040 and 0.024 ≤ ∇^2^ρ ≤ 0.139) for an interaction to be considered a hydrogen bond.^65^

From the above results, two features can be highlighted. (1) C–H···S interactions are found in both disulfides, which very likely influence the geometric properties and, consequently, the energetic properties of both compounds. (2) From a theoretical perspective, a good, but unusual, four-center six-electron O···S–S···O interaction is formed in 2DNDPDS.

#### 4.5.4 Theoretical Remarks

In the current section (Section 4.5), we have analyzed several weak intramolecular interactions, all of which intuitively contribute to the overall electronic energies of both disulfides. As the reader might guess, we have selected the interactions that we consider to be more affected by the isomerization 2DNDPDS → 4DNDPDS, and we have assumed that the remaining interactions should not change significantly. On the other hand, as we saw in Section 4.4, 4DNDPDS is more stable than 2DNDPDS. A natural question arises here: can the greater stability of 4DNDPDS be at least partially explained through the studied interactions? We believe it can be, as follows. Let us consider the stabilizing interactions (such as the electron-acceptor character of the −NO_2_ group) to decrease the total energy and the repulsive interactions to increase it. Then, we can sum the *E*(2) energies depicted in Figures 4–6 with the appropriate signs (i.e., negative if the contribution is stabilizing and positive otherwise), which renders a total of −352 and −349 kJ·mol^–1^ for 2DNDPDS and 4DNDPDS, respectively. This implies that the total energy contributions of the selected weak intramolecular interactions are almost equivalent in both disulfides. On the other hand, the BDE of the S–S bond in 4DNDPDS is 41.5 kJ·mol^–1^ larger than that in 2DNDPDS. Unfortunately, to the best of our knowledge, there is no universally accepted method to translate BDEs to stabilizing or destabilizing energies within the molecule. Nevertheless, we may conjecture that the rearrangement of intramolecular interactions (from a hypervalent 4c–6e interaction, the steric repulsion of the *n*_(O)_)(*n*_(S)_ interaction, a weaker electron delocalization between −NO_2_ and aryl moieties, stronger C–H···S hydrogen bonds, and a weaker S–S bond to a stronger electron delocalization between −NO_2_ and aryl moieties, weaker C–H···S hydrogen bonds, and a stronger S–S bond) is the main contributor to lowering the total electron energy of 4DNDPDS (by ∼−14.7 kJ·mol^–1^, see captions of Tables S9 and S10 of the Supporting Information for electron energies), which is consistent with the observed isomerization enthalpy [(−21.2 ± 5.4) kJ·mol^–1^].

To close this section, we recall that disulfide bonds, and how they acquire different conformations, might contribute to proper cell functioning via their role in the early stages of the protein folding process (e.g., see refs (66) and (67) and references therein); hence, the theoretical characterization presented here might be of interest to biologists and physical chemists.

## 5 Conclusions

We carried out an experimental and theoretical thermochemical study of 2,2′-dinitrodiphenyl disulfide (2DNDPDS) and 4,4′-dinitrodiphenyl disulfide (4DNDPDS), using differential scanning calorimetry, combustion calorimetry, thermogravimetry, molecular orbital calculations, natural bond orbital (NBO) analysis, and quantum theory of atoms in molecules (QTAIM) analysis. By means of calorimetric techniques and the G4 composite method together with atomization reactions, we obtained standard molar enthalpies of formation in the gas phase. Experimental and theoretical values were compared; we found differences < 5.5 kJ·mol^–1^.

The NBO analysis revealed a fascinating interaction in 2DNDPDS, namely, a hypervalent four-center six-electron O···S–S···O bond, which does not frequently occur in nature. In addition, the lowest-energy conformation of 2DNDPDS allows the formation of weak C–H···S hydrogen bonds. Both interactions counteract the intuitively expected steric repulsion between (−NO_2_) and sulfur’s lone electron pairs, as well as the delocalization loss caused by the (−NO_2_) non-coplanarity with the aryl ring. The stabilizing effects mentioned above, in conjunction with the strengthening of the S–S bond, are the main contributors to the observed isomerization enthalpy from 2DNDPDS to 4DNDPDS of (−21.2 ± 5.4) kJ·mol^–1^.

Finally, inserting two nitro groups at position 2 or 4 of the diphenyl disulfide structure is an exothermic process, which has an energy of (−31.4 ± 4.3) kJ·mol^–1^ or (−52.6 ± 5.5) kJ·mol^–1^, respectively.

## Supporting Information Available

Data of molar fractions, fusion temperatures, and enthalpies of fusion; values of heat capacity of the solid, liquid, and gaseous phases as a function of temperature and their respective fits; details of combustion experiments; details of thermogravimetric experiments; additional geometric parameters (from additional sources); optimized structures of 2DNDPDDS and 4DNDPDS at the level of MP2(full)/cc-pVDZ theory; and optimized structures of 2NPS and 4NPS, i.e., free radicals used to compute bond dissociation energies of S–S bonds, at the level of MP2(full)/cc-pVDZ theory (PDF)
