# Supplementary material for: Stabilizing Effect of a 4c/6e Hypervalent Bond in Dinitrodiphenyl Disulfides and Their Thermochemical Properties: Experimental and Computational Approach
Source: J Phys Chem A. 2023 Jun 27;127(26):5534–46. doi: 10.1021/acs.jpca.3c01624 (PMC10331731; doi:10.1021/acs.jpca.3c01624)
Supplement: Supplementary file 1 — jp3c01624_si_001.pdf [file jp3c01624_si_001.pdf]

Supplementary Material for:

The stabilizing effect of a 4c/6e hypervalent bond in dinitrodiphenyl disulfides and their thermochemical properties: Experimental and computational approach

Henoc Flores[*†], Fernando Ramos[†], Julio M. Hernández-Pérez[*†], Juan M. Solano-Altamirano[†], E. Adriana Camarillo[†], Jacinto Sandoval-Lira[‡]

[†]Facultad de Ciencias Químicas, Benemérita Universidad Autónoma de Puebla 14 sur y Av. San Claudio, C.P. 72570, Puebla Pue., México

[‡] Departamento de Ingeniería Ambiental, Instituto Tecnológico Superior de San Martín Texmelucan, C.P. 74120, San Martín Texmelucan, Pue., México

*Corresponding authors E–mail: henoc.flores@correo.buap.mx; julio.hernandez@correo.buap.mx

CONTENT **Page**

Table S1. Fusion experiments of the 2,2´-dinitrodiphenyl disulfide (2DNDPDS) and 4,4´-dinitrodiphenyl disulfide (4DNDPDS) from DSC determinations, at p°=0.1 MPa. **S3**

Table S2. Heat capacity data of the solid and liquid phases as temperature functions determined by DSC, for 2DNDPDS and 4DNDPDS. **S4**

Table S3. Heat capacity data of the gaseous phase as a temperature function, for 2DNDPDS and 4DNDPDS. **S5**

Table S4. Experiments of combustion of 2DNDPDS at p° = 0.1 MPa and T = 298.15 K. **S6**

Table S5. Experiments of combustion of 4DNDPDS at p° = 0.1 MPa y T = 298.15 K. **S6**

Table S6. Experimental data and calculated vaporization enthalpies for 2DNDPDS determined by thermogravimetry. **S8**

Table S7. Experimental data and calculated vaporization enthalpies for 4DNDPDS determined by thermogravimetry. **S9**

Table S8. Relevant geometric parameters of the optimized structures at the MP2(FULL)/cc-pVDZ level of theory and x-ray structures of 2DNDPDS and 4DNDPDS. **S10**

Table S9. XYZ coordinates of 2DNDPDS, as obtained from the optimization calculation at the MP2(full)/cc-pVDZ level of theory. **S10**

Table S10. XYZ coordinates of 4DNDPDS, as obtained from the optimization calculation at the MP2(full)/cc-pVDZ level of theory. **S11**

Table S11. XYZ coordinates of structure 2NPS radical, as obtained from the optimization calculation at the MP2(full)/cc-pVDZ level of theory. **S12**

Table S12. XYZ coordinates of structure 4NPS radical, as obtained from the optimization calculation at the MP2(full)/cc-pVDZ level of theory. **S13**

References **S14**

Table S1. Fusion experiments of the 2,2′-dinitrodiphenyl disulfide (2DNDPDS) and 4,4′-dinitrodiphenyl disulfide (4DNDPDS) from DSC determinations, at p°=0.1 MPa.

m_{sample}[a] / mg	purity / mole fraction	T_{fus} / K	$\Delta_{cr}^{l}H_{m}^{\circ}(T_{fus})$ / kJ · mol^{-1}
	2DNDPDS(s)		
3.3446	0.9995	470.77	39.80
2.8488	0.9998	470.57	39.45
2.4097	0.9995	470.86	39.44
3.5786	0.9997	470.62	39.11
	0.9996 ± 0.0002[b]	470.71 ± 0.38 [b]	39.45 ± 0.55 [b]
	4DNDPDS(s)		
4.4388	0.9991	456.03	36.09
7.4334	0.9991	456.51	35.28
2.3312	0.9989	456.11	36.20
4.0329	0.9988	456.28	35.65
	0.9990 ± 0.0002 [b]	456.23 ± 0.46 [b]	35.81 ± 0.75 [b]

[a]Standard uncertainties $u(m_{sample})$=0.0001 mg.
[b]Expanded uncertainties with a level of confidence of 0.95 and a coverage factor k=3.18, considering a t-student distribution, which include the contributions from the calibration.

Table S2. Heat capacity data of the solid and liquid phases as temperature functions determined by DSC, for 2DNDPDS and 4DNDPDS.

2DNDPDS (s)				2DNDPDS (l)	
$\frac{T}{\mathrm{K}}$	$\frac{C_p\ (\mathrm{s})}{\mathrm{J{\cdot}mol^{-1}{\cdot}K^{-1}}}$	$\frac{T}{\mathrm{K}}$	$\frac{C_p\ (\mathrm{s})}{\mathrm{J{\cdot}mol^{-1}{\cdot}K^{-1}}}$	$\frac{T}{\mathrm{K}}$	$\frac{C_p\ (\mathrm{l})}{\mathrm{J{\cdot}mol^{-1}{\cdot}K^{-1}}}$
283.15	300.3	373.15	387.5	485.15	566.4
288.15	306.2	378.15	390.5	488.15	572.4
293.15	309.9	383.15	395.7	491.15	581.4
298.15	316.8	388.15	399.7	494.15	590.6
303.15	321.3	393.15	403.4	497.15	600.2
308.15	327.0	398.15	408.2	500.15	602.5
313.15	331.3	403.15	411.9	503.15	605.5
318.15	335.6	408.15	416.4		
323.15	341.1	413.15	420.9		
328.15	346.3	418.15	425.0		
333.15	350.1	423.15	429.7		
338.15	354.7	428.15	433.3		
343.15	360.1	433.15	438.2		
348.15	363.8	438.15	441.8		
353.15	367.7	443.15	446.1		
358.15	372.3	448.15	451.2		
363.15	377.5	453.15	455.9		
368.15	382.2				

C_p(2NDFDS, s)/J·mol^{-1}·K^{-1}= $-250.2273+3.1898\ T/\mathrm{K} - 5.7194{\cdot}10^{-3}\ (T/\mathrm{K})^2 + 0.4671{\cdot}10^{-5}(T/\mathrm{K})^3$; r^2=0.9999

C_p(2NDFDS, l)/J·mol^{-1}·K^{-1}= $1200.2827{\cdot}10^{3}-7320.4900\ T/\mathrm{K} + 14.8834\ (T/\mathrm{K})^2 - 10.0823{\cdot}10^{-3}\ (T/\mathrm{K})^3$; r^2=0.9965

4DNDPDS (s)				4DNDPDS (l)	
$\frac{T}{\mathrm{K}}$	$\frac{C_p\ (\mathrm{s})}{\mathrm{J{\cdot}mol^{-1}{\cdot}K^{-1}}}$ [a]	$\frac{T}{\mathrm{K}}$	$\frac{C_p\ (\mathrm{s})}{\mathrm{J{\cdot}mol^{-1}{\cdot}K^{-1}}}$ [a]	$\frac{T}{\mathrm{K}}$	$\frac{C_p\ (\mathrm{l})}{\mathrm{J{\cdot}mol^{-1}{\cdot}K^{-1}}}$
288.15	302.1	368.15	357.2	473.15	532.9
293.15	302.7	373.15	362.5	476.15	541.4
298.15	303.5	378.15	366.4	479.15	549.2
303.15	305.1	383.15	371.3	482.15	556.6
308.15	307.7	388.15	376.7	485.15	563.7
313.15	309.5	393.15	381.7	488.15	569.6
318.15	311.7	398.15	387.2	491.15	576.1
323.15	315.9	403.15	392.6	494.15	580.4
328.15	319.8	408.15	401.1	497.15	583.9
333.15	323.0	413.15	407.7	500.15	587.2
338.15	327.6	418.15	414.5	503.15	588.6
343.15	332.9	423.15	421.5	506.15	588.6
348.15	337.2	428.15	428.6		
353.15	342.0	433.15	436.0		
358.15	346.7	438.15	443.5		
363.15	351.6	443.15	451.2		

C_p(4NDFDS, s)/J·mol^{-1}·K^{-1}= $775.9189-4.1127\ T/\mathrm{K} + 10.2484{\cdot}10^{-3}\ (T/\mathrm{K})^2 - 0.5926{\cdot}10^{-5}(T/\mathrm{K})^3$; r^2=0.9993

C_p(4NDFDS, l)/J·mol^{-1}·K^{-1}= $71679.9206-461.1313\ T/\mathrm{K} +989.9387{\cdot}10^{-3}\ (T/\mathrm{K})^2 + 70.4097{\cdot}10^{-5}(T/\mathrm{K})^3$; r^2=0.9998

Standard uncertainty $u(T)$=0.01 K. Regarding compounds in solid and liquid phases, the uncertainty corresponds to $U(C_p$, 2DNDPDS, s)= 6.0 J·mol^{-1}·K^{-1}, $U(C_p$, 2DNDPDS, l)= 7.0 J·mol^{-1}·K^{-1}, $U(C_p$, 4DNDPDS, s)= 4.6 J·mol^{-1}·K^{-1}, $U(C_p$, 4DNDPDS, l)= 9.0 J·mol^{-1}·K^{-1} with a level of confidence of 0.95 and a cover factor k=2.26 assuming a t-student distribution.

Table S3. Heat capacity data of the gaseous phase as a temperature function, for 2DNDPDS and 4DNDPDS.

$\frac{T}{\text{K}}$	$\frac{C_p\text{ (g)}}{\text{J·mol}^{-1}\text{·K}^{-1}}$ 2NDFDS (g)	4NDFDS(g)
298.15	276.62	277.39
300.00	278.00	278.77
310.00	285.44	286.17
320.00	292.78	293.47
330.00	300.02	300.68
340.00	307.15	307.77
350.00	314.17	314.75
360.00	321.06	321.62
370.00	327.84	328.36
380.00	334.48	334.98
390.00	341.00	341.47
400.00	347.39	347.82
410.00	353.64	354.05
420.00	359.77	360.15
430.00	365.76	366.11
440.00	371.61	371.95
450.00	377.34	377.65
460.00	382.93	383.22
470.00	388.40	388.66
480.00	393.74	393.98
490.00	398.95	399.17
500.00	404.03	404.23

Table S4. Experiments of combustion of 2DNDPDS at $p°$ = 0.1 MPa and T = 298.15 K.

m(Compound) / g	0.46793	0.44618	0.46714	0.41584	0.46588	0.45670	0.44025
m'(Cotton) / g	0.01293	0.01389	0.01235	0.01372	0.01343	0.01353	0.01298
m''(Benzoic acid) / g	0.50640	0.51668	0.49624	0.50357	0.53864	0.55292	0.55371
m'''(Pt) / g	6.13110	6.13110	6.13986	6.13776	6.15041	6.14773	6.16044
$p(O_2)$ / MPa	3.04	3.04	3.04	3.04	3.04	3.04	3.04
T_i / K	296.20431	296.20450	296.20402	296.20383	296.21370	296.21031	296.22340
T_f / K	297.92381	297.90830	297.90501	297.83926	297.99095	297.99932	297.98510
ΔT_c / K	1.70288	1.68650	1.68260	1.61523	1.75835	1.77092	1.74520
ε_i / J·K^{-1}	53.58	53.58	53.57	53.53	53.62	53.63	53.62
ε_f / J·K^{-1}	53.90	53.94	53.88	53.86	54.00	54.01	54.00
$\Delta_{ign}u$ / J	4.2	4.2	4.2	4.2	4.2	4.2	4.2
$-m'\cdot\Delta_c u'$ / J	219.22	235.49	209.38	232.61	227.69	229.39	220.06
$-m''\cdot\Delta_c u''$ / J	13376.05	13647.59	13107.68	13301.30	14227.64	14604.83	14625.70
ε(calor)·$(-\Delta T_c)$ / J	-24457.95	-24222.69	-24166.68	-23199.06	-25254.65	-25435.19	-25065.78
$\varepsilon_{cont}\cdot(-\Delta T_c)$ / J	-91.17	-90.26	-90.06	-86.35	-94.22	-94.91	-93.50
$\Delta_{IBP}u$ / J	-24544.93	-24308.76	-24252.54	-23281.22	-25344.68	-25525.91	-25155.09
$\Delta u(HNO_3)$ / J	99.70	82.98	100.30	91.34	90.15	94.92	91.34
Δu_{corr} / J	30.33	30.60	29.91	29.46	31.67	32.01	31.79
$\Delta_c u°$ /J·g^{-1}	-23122.3	-23112.0	-23130.7	-23149.5	-23112.2	-23132.8	-23137.3

⟨$-\Delta_c u°$ (298.15 K)/J·g^{-1}⟩ = 23128.1 ± 5.2

The uncertainties associated with each average specific combustion energy is the standard uncertainty of seven experiments.

Table S5. Experiments of combustion of 4DNDPDS at $p°$ = 0.1 MPa y T = 298.15 K.

m(Compound) / g	0.40153	0.38943	0.39560	0.40369	0.39997	0.37717	0.39027
m'(Cotton) / g	0.01308	0.01293	0.01257	0.01247	0.01244	0.01287	0.01257
m''(Benzoic acid) / g	0.66230	0.66537	0.68262	0.69417	0.68977	0.69842	0.71894
m'''(Pt) / g	6.11876	6.12297	6.13278	6.12272	6.13581	6.12449	6.12765
$p(O_2)$ / MPa	3.04	3.04	3.04	3.04	3.04	3.04	3.04
T_i / K	296.20420	296.20511	296.20782	296.17769	296.17591	296.17521	296.15714
T_f / K	298.10090	298.08620	298.13151	298.13559	298.11882	298.09508	298.13813
ΔT_c / K	1.87970	1.86478	1.90798	1.94110	1.92691	1.90514	1.96528
ε_i / J·K^{-1}	53.69	53.68	53.71	53.73	53.72	53.71	53.74
ε_f / J·K^{-1}	54.22	54.22	54.26	54.30	54.28	54.31	54.34
$\Delta_{ign}u$ / J	4.2	4.2	4.2	4.2	4.2	4.2	4.2
$-m'\cdot\Delta_c u'$ / J	221.76	219.22	213.11	211.42	210.91	218.20	213.11
$-m''\cdot\Delta_c u''$ / J	17493.99	17575.08	18030.72	18335.81	18219.58	18448.07	18990.08
ε(calor)·$(-\Delta T_c)$ / J	-26997.57	-26783.28	-27403.74	-27879.44	-27675.63	-27362.95	-28226.73
$\varepsilon_{cont}\cdot(-\Delta T_c)$ / J	-100.88	-100.05	-102.45	-104.27	-103.48	-102.28	-105.60
$\Delta_{IBP}u$ / J	-27094.25	-26879.13	-27501.99	-27979.51	-27774.91	-27461.03	-28328.13
$\Delta u(HNO_3)$ / J	85.37	80.59	85.07	83.58	82.98	71.64	82.39
Δu_{corr} / J	35.40	35.36	36.08	36.69	36.46	36.54	37.44
$\Delta_c u°$ /J·g^{-1}	-23056.1	-23030.8	-23096.6	-23067.2	-23064.2	-23031.0	-23074.1

⟨$-\Delta_c u°$ (298.15 K)/J·g^{-1}⟩ = 23060.0 ± 8.9

The uncertainties associated with each average specific combustion energy is the standard uncertainty of seven experiments.

In tables S4 and S5: m (compound) is the mass of the compound;, m' (Cotton) is the mass of cotton; m''(Benzoic acid) is the mass of benzoic acid; m'''(Pt) is the mass of Platinum; $p(O_2)$ is the initial pressure; T_i and T_f are the initial and final temperatures of the main period of the combustion experiment; ΔT_c is the corrected temperature increase; ε^i and ε^f, are the equivalent energies of the contents of the bomb in the initial and final state respectively; $\Delta_{ign}u$, is the energy supplied to the

sample for ignition; $m' \cdot \Delta_c u'$ is the energy of combustion of cotton; $m'' \cdot \Delta_c u''$ is the energy of combustion of benzoic acid; ε(calor) is the energy equivalent of the calorimeter; ε_{cont} $(-\Delta T_c) = \varepsilon^i (T_i - 298.15 \text{ K}) + \varepsilon^f (298.15 \text{ K} - T_i + \Delta T_{corr})$, where ΔT_{corr} is the temperature correction for the heat of stirring and the heat exchange between the calorimeter and its environment; $\Delta_{IBP}u$ is the energy of the isothermal process of the bomb; Δu (HNO_3) is the decomposition energy of nitric acid; Δu_{corr} is the correction to the standard state which includes the dilution energy of sulfuric acid; and $\Delta_c u°$ is the specific energy of combustion. These values were calculated according to the NIST recommendations (https://trc.nist.gov/cctool/) [1]. 10 cm^3 of deionized water were used in these combustion experiments. The reported masses were corrected for buoyancy.

Table S6. Experimental data and calculated vaporization enthalpies for 2DNDPDS determined by thermogravimetry.

$\frac{T}{\text{K}}$	$\frac{m}{\text{mg}}$	$\frac{(\text{d}m/\text{d}t)\cdot 10^9}{\text{kg}\cdot\text{s}^{-1}}$	$\frac{(1/T)\cdot 10^3}{\text{K}^{-1}}$	$\ln(\text{d}m/\text{d}t\cdot T)$
**Series 1**				
480.0	25.7697	1.4869	2.083	-14.153
482.0	25.7497	1.6239	2.075	-14.060
484.0	25.7278	1.7803	2.066	-13.964
486.0	25.7042	1.9581	2.058	-13.865
488.0	25.6792	2.1276	2.049	-13.778
490.0	25.6521	2.3373	2.041	-13.680
492.0	25.6226	2.5593	2.033	-13.585
494.0	25.5907	2.7791	2.024	-13.499
496.0	25.5556	3.0463	2.016	-13.403
498.0	25.5175	3.3102	2.008	-13.316
500.0	25.4760	3.6073	2.000	-13.226
Series 1 $\ln(\text{d}m/\text{d}t\ T)$=9.1–11156.4/$T$; r^2=0.9999; σ_a=0.1; σ_b=28.9; $\Delta_l^g H_m$(490.0 K)/kJ·mol^{-1}=92.8 ± 0.2				
**Series 2**				
480.0	9.5479	1.3612	2.083	-14.241
482.0	9.5309	1.4530	2.075	-14.172
484.0	9.5129	1.5559	2.066	-14.099
486.0	9.4932	1.6843	2.058	-14.016
488.0	9.4717	1.8887	2.049	-13.897
490.0	9.4481	2.0545	2.041	-13.809
492.0	9.4220	2.2630	2.033	-13.708
494.0	9.3930	2.5245	2.024	-13.595
496.0	9.3626	2.6950	2.016	-13.525
498.0	9.3287	2.9268	2.008	-13.439
500.0	9.2912	3.2126	2.000	-13.342
Series 2 $\ln(\text{d}m/\text{d}t\ T)$=8.9–11105.3/$T$; r^2=0.9971; σ_a=0.4; σ_b=200.8; $\Delta_l^g H_m$(490.0 K)/kJ·mol^{-1}=90.3± 1.7				
**Series 3**				
480.0	11.1066	1.3908	2.083	-14.220
482.0	11.0894	1.4703	2.075	-14.160
484.0	11.0713	1.5707	2.066	-14.090
486.0	11.0508	1.7661	2.058	-13.968
488.0	11.0290	1.8783	2.049	-13.903
490.0	11.0049	2.0906	2.041	-13.791
492.0	10.9790	2.2866	2.033	-13.698
494.0	10.9499	2.4905	2.024	-13.608
496.0	10.9182	2.7122	2.016	-13.519
498.0	10.8839	2.9918	2.008	-13.417
500.0	10.8463	3.2763	2.000	-13.322
Series 3 $\ln(\text{d}m/\text{d}t\ T)$=8.7–11012.1/$T$; r^2=0.9970; σ_a=0.4; σ_b=201.4; $\Delta_l^g H_m$(490.0 K)/kJ·mol^{-1}=91.6± 1.7				
**Series 4**				
480.0	10.9214	1.4758	2.083	-14.160
482.0	10.9023	1.6296	2.075	-14.057
484.0	10.8819	1.7636	2.066	-13.974
486.0	10.8595	1.9459	2.058	-13.871
488.0	10.8350	2.1223	2.049	-13.780
490.0	10.8081	2.3363	2.041	-13.680
492.0	10.7790	2.5509	2.033	-13.588
494.0	10.7468	2.8018	2.024	-13.490
496.0	10.7119	3.0239	2.016	-13.410
498.0	10.6737	3.3178	2.008	-13.313
500.0	10.6326	3.5862	2.000	-13.232
Series 4 $\ln(\text{d}m/\text{d}t\ T)$=9.2–11197.8/$T$; r^2=0.9998; σ_a=0.1; σ_b=56.3; $\Delta_l^g H_m$(490.0 K)/kJ·mol^{-1}=93.1± 0.5				
$\langle\Delta_l^g H_m$(2DNDPDS, 490.0 K)$\rangle$/kJ·mol^{-1}= 92.8 ± 0.4				

Table S7. Experimental data and calculated vaporization enthalpies for 4DNDPDS determined by thermogravimetry.

$\frac{T}{\mathrm{K}}$	$\frac{m}{\mathrm{mg}}$	$\frac{(\mathrm{d}m/\mathrm{d}t)\cdot 10^9}{\mathrm{kg\cdot s^{-1}}}$	$\frac{(1/T)\cdot 10^3}{\mathrm{K^{-1}}}$	$\ln(\mathrm{d}m/\mathrm{d}t\cdot T)$
**Series 1**				
470.0	14.3038	0.5095	2.128	-15.245
472.0	14.2976	0.5544	2.119	-15.156
474.0	14.2905	0.6251	2.110	-15.032
476.0	14.2826	0.6950	2.101	-14.922
478.0	14.2740	0.7505	2.092	-14.841
480.0	14.2643	0.8557	2.083	-14.705
482.0	14.2538	0.9034	2.075	-14.647
484.0	14.2422	1.0580	2.066	-14.485
486.0	14.2293	1.1177	2.058	-14.426
488.0	14.2152	1.2205	2.049	-14.334
490.0	14.1997	1.3533	2.041	-14.226
Series 1 $\ln(\mathrm{d}m/\mathrm{d}t\ T)=9.9–11798.7/T$; $r^2=0.9974$; $\sigma_a=0.4$; $\sigma_b=200.3$; $\Delta_l^g H_m$(480.0 K)/kJ·mol^{-1}=98.1 ± 1.7				
**Series 2**				
470.0	15.9355	0.4090	2.128	-15.464
472.0	15.9302	0.4675	2.119	-15.327
474.0	15.9245	0.5013	2.110	-15.253
476.0	15.9181	0.5605	2.101	-15.137
478.0	15.9109	0.6306	2.092	-15.015
480.0	15.9031	0.6843	2.083	-14.929
482.0	15.8943	0.7671	2.075	-14.810
484.0	15.8847	0.8413	2.066	-14.714
486.0	15.8741	0.9351	2.058	-14.604
488.0	15.8624	1.0316	2.049	-14.502
490.0	15.8494	1.1314	2.041	-14.405
Series 2 $\ln(\mathrm{d}m/\mathrm{d}t\ T)=10.4–12134.2/T$; $r^2=0.9992$; $\sigma_a=0.2$; $\sigma_b=112.9$; $\Delta_l^g H_m$(480.0 K)/kJ·mol^{-1}=100.9 ± 0.9				
Series 3				
470.0	18.1299	0.4188	2.128	-15.441
472.0	18.1250	0.4980	2.119	-15.264
474.0	18.1185	0.5493	2.110	-15.161
476.0	18.1117	0.5895	2.101	-15.086
478.0	18.1041	0.6709	2.092	-14.953
480.0	18.0957	0.7489	2.083	-14.839
482.0	18.0865	0.8023	2.075	-14.766
484.0	18.0764	0.8858	2.066	-14.662
486.0	18.0651	0.9724	2.058	-14.565
488.0	18.0528	1.0652	2.049	-14.470
490.0	18.0396	1.1512	2.041	-14.388
Series 3 $\ln(\mathrm{d}m/\mathrm{d}t\ T)=9.7–11797.5/T$; $r^2=0.9956$; $\sigma_a=0.5$; $\sigma_b=261.0$; $\Delta_l^g H_m$(480.0 K)/kJ·mol^{-1}=98.1± 2.2				
**Series 4**				
470.0	18.1950	0.4004	2.128	-15.486
472.0	18.1898	0.4584	2.119	-15.346
474.0	18.1838	0.5039	2.110	-15.247
476.0	18.1776	0.5317	2.101	-15.189
478.0	18.1708	0.5909	2.092	-15.080
480.0	18.1634	0.6605	2.083	-14.964
482.0	18.1549	0.7452	2.075	-14.839
484.0	18.1454	0.8240	2.066	-14.735
486.0	18.1350	0.9248	2.058	-14.615
488.0	18.1233	1.0166	2.049	-14.516
490.0	18.1106	1.1090	2.041	-14.425
Series 4 $\ln(\mathrm{d}m/\mathrm{d}t\ T)=10.5–12211.0/T$; $r^2=0.9970$; $\sigma_a=0.5$; $\sigma_b=224.7$; $\Delta_l^g H_m$(480.0 K)/kJ·mol^{-1}=101.5± 1.9				
<$\Delta_l^g H_m$(4DNDPDS, 480.0 K)>/kJ·mol^{-1}= 100.2 ± 1.4				

Standard uncertainties u are $u(T) = 0.1$ K, $u(m) = 0.1$ µg, and the combined expanded uncertainty U_c is $U_c(dm/dt) = 0.066 \cdot 10^9$ kg·s^{-1}, $U_c(1/T) = 0.001$ x10^3 K^{-1}, $U_c(\ln(dm/dt \cdot T^{1/2})$=0.020; $U_c(\ln(dm/dt \cdot T)$=0.020 (0.95 level of confidence).

Table S8. Relevant geometric parameters of the optimized structures at the MP2(FULL)/cc-pVDZ level of theory and x-ray structures of 2DNDPDS and 4DNDPDS.

Parameters	2DNDPDS				4DNDPDS		
	MP2	[Ref. 34]	[Ref. 2]	[Ref. 3]	MP2	[Ref. 15]	[Ref. 4]
S1 - S2 (Å)	2.090	2.045	2.057	2.058	2.065	2.019	2.029
S1 - C1 (Å)	1.79	1.785	1.784	1.793	1.790	1.767	1.779
S2 - C7 (Å)	1.799	1.808	1.795	1.791	1.790	1.767	1.779
S2 - S1 – C1 (°)	102.4	104.2	105.1	105.8	104.0	106.2	105.7
S1 - S2 – C7 (°)	102.4	104.6	104.4	106.0	104.0	106.2	105.7
C1 – S1 – S2 - C7 (°)	86.4	85.1	-84.4	-85.5	84.6	90.1	88.1
S2 - S1 – C1 – C2 (°)	-15.6	-19.1	11.53	16.7	-15.7	-21.7	-22.0
S2 - S1 – C7 – C8 (°)	-15.7	-10.8	20.0	19.1	-15.7	-21.7	-22.0

Refs. [34] and [15] of the Manuscript, Refs. [2], [3], and [4] of the Supplementary Material. After optimization, all geometries rendered the same optimized geometries.

Table S9. XYZ coordinates of 2DNDPDS, as obtained from the optimization calculation at the MP2(full)/cc-pVDZ level of theory.

Energy + ZPE: -1665.082546 ua

Atom	X	Y	Z
6	-1.847353000	0.006707000	-0.288606000
6	3.387054000	2.029777000	1.582915000
6	2.002349000	1.938469000	1.795407000
6	1.249517000	0.943014000	1.160368000
6	-3.244031000	0.123055000	-0.099226000
6	-4.009728000	1.111253000	-0.733055000
6	-3.386789000	2.030051000	-1.582917000
6	-2.002035000	1.938801000	-1.795106000
6	-1.249306000	0.943333000	-1.159960000
6	1.847397000	0.006472000	0.288816000
6	3.244030000	0.122871000	0.099109000
6	4.009818000	1.111089000	0.732817000
1	3.977867000	2.806992000	2.074482000
1	1.496587000	2.645218000	2.459675000

1	0.177742000	0.873198000	1.358456000
1	-5.084943000	1.130918000	-0.547649000
1	-3.977522000	2.807281000	-2.074579000
1	-1.496139000	2.645628000	-2.459187000
1	-0.177499000	0.873543000	-1.357838000
1	5.084982000	1.130855000	0.547108000
7	-3.966925000	-0.796541000	0.793817000
7	3.966787000	-0.796593000	-0.794151000
8	-3.288575000	-1.411844000	1.625230000
8	-5.187484000	-0.888749000	0.664867000
8	3.288186000	-1.412426000	-1.624977000
8	5.187477000	-0.888161000	-0.665931000
16	-0.894931000	-1.274323000	0.540650000
16	0.894776000	-1.274527000	-0.540339000

Table S10. XYZ coordinates of 4DNDPDS, as obtained from the optimization calculation at the MP2(full)/cc-pVDZ level of theory.

Energy + ZPE: -1665.088054 ua

Atom	X	Y	Z
16	0.559157000	0.868134000	2.485959000
6	-0.189223000	1.866086000	1.202158000
6	0.189223000	3.225019000	1.183965000
6	-0.300074000	4.083463000	0.193315000
6	-1.174662000	3.566330000	-0.769912000
7	-1.701341000	4.467268000	-1.816325000
8	-1.331790000	5.642253000	-1.786224000
8	-2.471721000	3.979740000	-2.644884000
6	-1.569391000	2.224767000	-0.768384000
6	-1.065753000	1.369063000	0.220737000
1	0.863715000	3.617097000	1.952140000
1	-0.022708000	5.138219000	0.163816000
1	-2.254452000	1.862492000	-1.536594000
1	-1.373359000	0.320449000	0.237155000

16	-0.559157000	-0.868134000	2.485959000
6	0.189223000	-1.866086000	1.202158000
6	-0.189223000	-3.225019000	1.183965000
6	0.300074000	-4.083463000	0.193315000
6	1.174662000	-3.566330000	-0.769912000
7	1.701341000	-4.467268000	-1.816325000
8	1.331790000	-5.642253000	-1.786224000
8	2.471721000	-3.979740000	-2.644884000
6	1.569391000	-2.224767000	-0.768384000
6	1.065753000	-1.369063000	0.220737000
1	-0.863715000	-3.617097000	1.952140000
1	0.022708000	-5.138219000	0.163816000
1	2.254452000	-1.862492000	-1.536594000
1	1.373359000	-0.320449000	0.237155000

Table S11. XYZ coordinates of structure 2DNDPDS radical, as obtained from the optimization calculation at the MP2(full)/cc-pVDZ level of theory.

Energy + ZPE: -832.495730 ua

Atom	X	Y	Z
6	-2.342263000	-1.116536000	-0.000169000
6	-2.646891000	0.257855000	0.000193000
6	-1.625320000	1.211239000	0.000290000
6	-0.261587000	0.825461000	0.000132000
6	0.006995000	-0.558528000	-0.000027000
6	-1.007368000	-1.527476000	-0.000202000
1	-3.141775000	-1.861672000	-0.000541000
1	-3.688822000	0.590904000	0.000350000
1	-1.874500000	2.276464000	0.000365000
1	-0.722484000	-2.580763000	-0.000344000
7	1.400656000	-1.014896000	0.000076000

8	2.277163000	-0.136770000	0.000344000
8	1.622730000	-2.225673000	-0.000028000
16	0.980153000	2.064175000	-0.000262000

Table S12. XYZ coordinates of structure 4DNDPDS radical, as obtained from the optimization calculation at the MP2(full)/cc-pVDZ level of theory.

Energy + ZPE: -832.490580 ua

Atom	X	Y	Z
16	-3.448936000	-0.000142000	-0.000257000
6	-1.719349000	0.000239000	0.000229000
6	-1.005871000	-1.227261000	-0.012862000
6	0.383048000	-1.214216000	0.004739000
6	1.086099000	-0.000001000	0.000167000
7	2.541743000	-0.000085000	0.000007000
8	3.114325000	-1.096081000	0.017160000
8	3.114420000	1.095835000	-0.017610000
6	0.383249000	1.214328000	-0.004379000
6	-1.005702000	1.227654000	0.013256000
1	-1.526321000	-2.190292000	-0.040909000
1	0.941944000	-2.152277000	0.000454000
1	0.942445000	2.152234000	-0.000106000
1	-1.526103000	2.190716000	0.041325000

References

[1] Paulechka, E.; Riccardi, D.; Bazyleva, A.; Ribeiro da Silva, M. D. M. C.; Zaitsau D. Corrections to standard state in combustion calorimetry: An update and a web-based tool. *J. Chem. Thermodyn*. **2021**, *158*, 106425.

[2] Glidewell, C.; Low, J. N.; Wardell, J. L. Conformational preferences and supramolecular aggregation in 2-nitrophenylthiolates: disulfides and thiosulfonates. *Acta Cryst.* **2000***, B56*, 893-905.

[3] Song, M.; Fan, C. 1,2-Bis(2-nitro-phenyl)disulfane. *Acta Cryst.* **2009**, *E65*, o2835.

[4] Wardell, J. L.; Low, J. N.; Glidewell, C. Bis(4-nitro-phenyl) di-sulfide at 150 K, a three-dimensional framework built from C-HO hydrogen bonds and aromatic stacking interactions. *Acta Cryst.* **2000**, *C56*, 679-681.
